# Global, regional, and national burden of pulmonary arterial hypertension in adults aged ≥60 years from 1990–2021: SDI-stratified trend analysis and projections to 2050

**DOI:** 10.3389/fmed.2025.1595504

**Published:** 2025-11-25

**Authors:** Guorui Xu, Xiangyi Feng, Nini Qu

**Affiliations:** ^1^The First Clinical College of Liaoning University of Traditional Chinese Medicine, Shenyang, Liaoning, China; ^2^Department of Pulmonology, The First Affiliated Hospital of Liaoning University of Traditional Chinese Medicine, Shenyang, Liaoning, China

**Keywords:** pulmonary arterial hypertension, Global Burden of Disease study, incidence, mortality, Sociodemographic Index

## Abstract

**Background:**

Pulmonary arterial hypertension (PAH), a progressive and life-threatening condition, has significant implications for public health, particularly in the aging population. This study provides a comprehensive analysis of PAH in individuals aged 60 years and older from 1990 to 2021, utilizing data from the Global Burden of Disease (GBD) 2021 study, which encompasses 204 countries and territories.

**Methods:**

We tracked PAH trends (1990–2021) with three metrics: the annual percentage change (APC) from joinpoint, the average APC (AAPC) across segments, and the estimated APC (EAPC) from the log-linear model. APC captures year-by-year shifts; AAPC summarises the whole period; EAPC gives a single long-term slope. Significance set at 95% CI excluding zero. For 2022–2050 we used the Bayesian age-period-cohort (BAPC) model, which extends these time-component ideas into future projections.

**Results:**

Between 1990 and 2021, the number of new cases increased by 130%, deaths by 130%, and disability-adjusted life years (DALYs) increased by 100%. In 2021, there were 15,622 new cases, 15,443 deaths, and 248,064 DALYs globally. The incidence rate rose from 1.447 to 1.66 per 100,000 population. Looking ahead to 2050, we project the number of new cases to reach 31383 (95% CI: 17604.40, 45162.56), an incidence rate of 1.46 per 100,000, approximately 20,687 deaths, and 302,760 DALYs. In addition, Zambia recorded the highest incidence rate, while China had the largest total number of cases. The middle Sociodemographic Index (SDI) region bore the highest disease burden, highlighting disparities in the global distribution of PAH.

**Conclusion:**

These findings underscore the escalating burden of PAH among the elderly and emphasize the urgent need for targeted public health strategies to address this growing challenge. Our study highlights the importance of continued surveillance and the development of interventions to mitigate the impact of PAH on aging populations worldwide.

## 1 Introduction

Pulmonary arterial hypertension (PAH) is a progressive disease characterized by increased pulmonary vascular resistance and altered right ventricular function and structure (1, 2). Patients with PAH typically experience shortness of breath, especially during activity, along with symptoms such as weakness, chest pain, swelling of the ankles or legs, and dizziness, which can eventually lead to death (3–6). Reportedly, approximately 1% of the global population suffers from PAH, which affects 10% of people over 65 years of age (7). Between 1990 and 2021, although the age-standardized incidence showed no significant change, the number of patients with PAH has increased by 81.5%, with a higher proportion in the elderly population (8). The Rotterdam Epidemiological Study of Hypertension reported higher incidence among elderly participants compared to younger patients (9). These findings indicate a growing disease burden of PAH in the elderly population, highlighting the need for greater attention to incidence and mortality trends among this demographic. However, comprehensive global studies focusing on the elderly remain limited, and systematic reports on incidence and mortality trends in elderly PAH patients are lacking.

The Global Burden of Disease (GBD) study, spearheaded by the Institute for Health Metrics and Evaluation (IHME) at the University of Washington, is widely recognized as one of the most comprehensive global epidemiological databases. It provides a robust framework for comparative assessments of morbidity and mortality across countries, regions, and globally, facilitating a nuanced understanding of disease distributions and trends (10, 11). Despite the significant advancements in our understanding of PAH distribution brought about by previous GBD studies, these investigations have predominantly focused on an overall perspective that encompasses all age groups (8). While these studies have documented global, regional, and national trends from 1990 to 2021, they are not without limitations. Specifically, data on the elderly population remain fragmented, and variations in methodology complicate cross-national comparisons and overall assessments. This fragmentation and methodological diversity hinder the development of a cohesive understanding of PAH’s impact on the elderly. Furthermore, existing research has insufficiently addressed the prediction of future disease burdens. This gap in predictive research introduces substantial uncertainty into addressing the healthcare demands arising from global population aging. As the global population continues to age, the need for accurate projections and targeted public health strategies becomes increasingly urgent.

This study aims to provide a comprehensive analysis of the epidemiological trends of PAH among adults aged 60 and older, utilizing data from the GBD database spanning from 1990 to 2021. The study will analyze global, regional, and national epidemiological data on PAH among adults aged 60 and older. Key disease burden indicators, including incidence rate, mortality rate, and disability-adjusted life years (DALYs), will be examined to identify detailed temporal and spatial trends. Additionally, building on established epidemiological data and trends, robust statistical projection models will be employed to forecast the incidence of PAH in this population through 2050. This study aims to support the development of targeted public health interventions and improve the management of PAH in aging populations globally.

## 2 Methods

### 2.1 Overview and methodological details

Data for this study were sourced from the GBD database, encompassing 204 countries and territories. In GBD 2021, PAH is classified under Group 1, characterized by pulmonary arterial remodelling, elevated pulmonary pressure, and eventual right ventricular dysfunction. Only records with ICD-10 codes 416 and I27.0 were included after chart review confirmed the diagnosis. Data coded I27.2 or in ICD-8, ICD-9-BTL, and ICD-10 tabular formats were excluded to ensure specificity, as they do not reliably distinguish PAH from other pulmonary disorders. In countries where the introduction of I27.0 caused abrupt changes in PAH mortality trends, pre-implementation years were omitted (11). This approach isolates PAH from other forms of pulmonary hypertension (PH) associated with left heart disease, lung diseases, hypoxia, pulmonary artery obstructions, and multifactorial mechanisms. The analysis covers multiple dimensions, including gender, age groups (60–64, 65–69, 70–74, 75–79, 80–84, 85–89, 90–94, and over 95 years), and location.

### 2.2 Sociodemographic Index

The Sociodemographic Index (SDI) is a composite indicator measuring a country or region’s socioeconomic development, incorporating factors such as economic level, education, health, social security, welfare, environment, and sustainability. The GBD database categorizes countries and regions into five SDI levels: low, low-middle, middle, high-middle, and high (12).

### 2.3 Statistical analysis

This study analyzed the incidence, mortality, and DALYs of PAH per 100,000 population, presenting estimates with 95% uncertainty intervals (UI) to quantify reliability. The 95% UI was calculated using the Bayesian hierarchical model employed by the GBD database, integrating uncertainties from multiple data sources (e.g., national registries, clinical studies, cohort studies) and estimating them via a Markov chain Monte Carlo (MCMC) approach, thereby reflecting estimation errors arising from data sources, diagnostic variations, model assumptions, and other factors.

To address potential data biases and varying age structures, we used the age-standardized rate (ASR) based on the GBD world standard population. Trends from 1990 to 2021 were assessed using multiple approaches: segmented regression (Joinpoint analysis) identified distinct temporal segments and calculated the annual percentage change (APC) with 95% confidence intervals (CI) for each segment; the average annual percentage change (AAPC) summarized the overall long-term trend; and a log-linear model estimated the average annual percentage change (EAPC) for sustained, long-term trends, providing a single summary slope for direct comparison of 1990–2021 trajectories across regions, countries, or diseases. For all measures (APC, AAPC, EAPC), trends were considered statistically significant if the 95% CI did not include zero (*p* < 0.05) (13). The association between PAH burden and the SDI was examined through curve fitting. In regional and national stratified analyses, the statistical significance of between-group differences was assessed by examining the overlap of UI, with non-overlapping UI suggesting significant differences.

To project the disease burden from 2022 to 2050, we employed the Bayesian age-period-cohort (BAPC) model. This model disentangles the independent effects of age, period, and cohort, making it suitable for long-term forecasting. The model was configured with second-order random walk priors for age, period, and cohort effects to ensure smoothness and identifiability (14, 15). The population aged 60 and above was divided into eight 5-year age groups: 60–64, 65–69, 70–74, 75–79, 80–84, 85–89, 90–94, and 95+.

The BAPC model was fitted using the integrated nested Laplace approximation (INLA) algorithm. Projections assume no major public health disruptions (e.g., pandemics, wars) and stable socioeconomic trends, indicating that current disease rates and population structures will persist into the future without significant external shocks.

The analyses, including the BAPC modelling, were conducted using R software (version 4.4.2) with packages such as BAPC and INLA. A two-tailed *p*-value of less than 0.05 was considered statistically significant.

### 2.4 Age-standardized rates

The proportion of people over 60 years of age based on the standard population is calculated and displayed in the “Age Distribution of Std Pop” column. These will be used as weights.

## 3 Results

### 3.1 Global burden of PAH in older adults (≥60 years)

#### 3.1.1 Incidence

**Figure 1:**
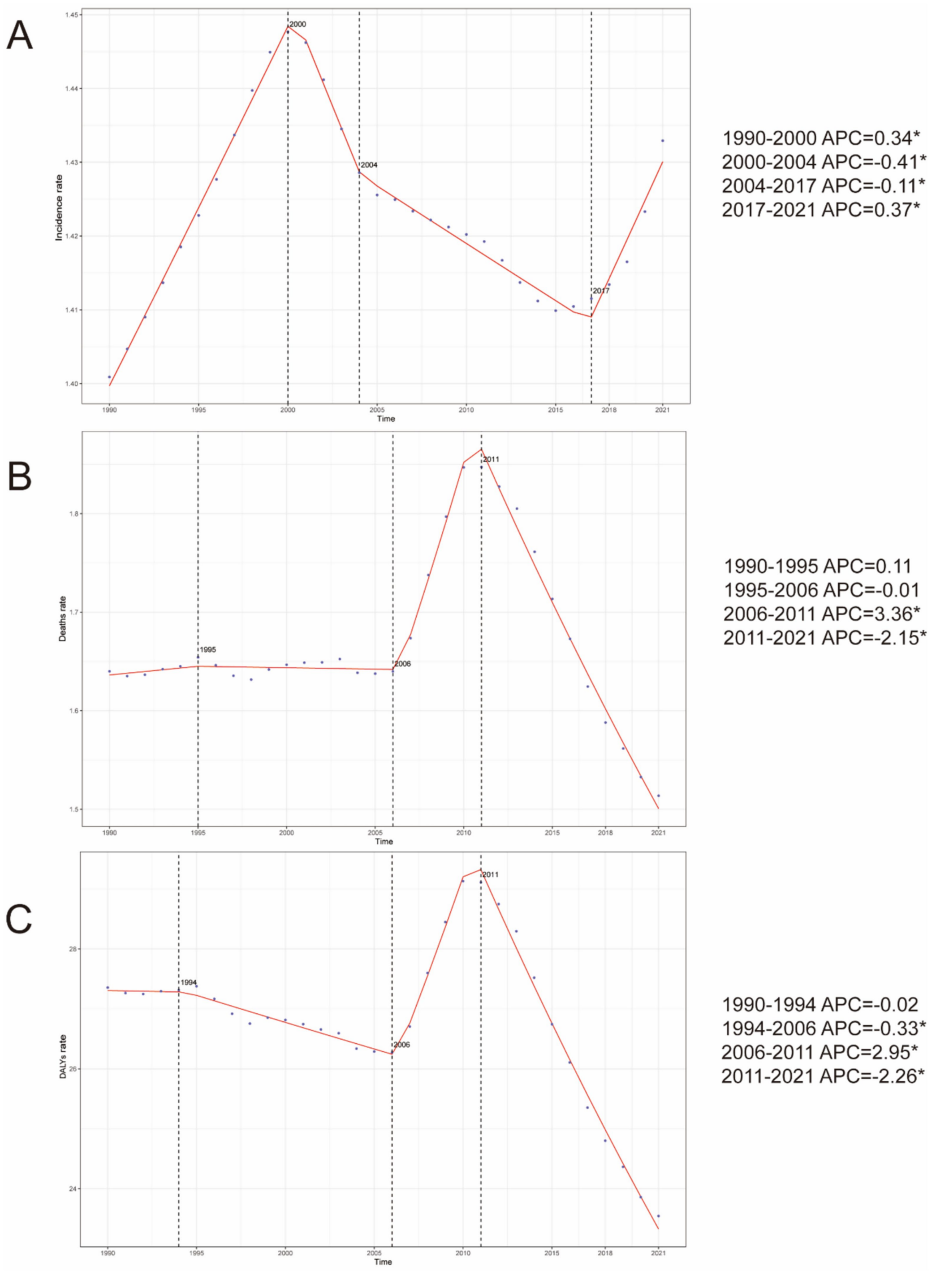
Annual percent change (APC) in PAH incidence, mortality, and DALYs in older adults globally (DALYs) from 1990 to 2021. **(A)** Incidence. **(B)** Mortality. **(C)** DALYs rate.

**Figure 2:**
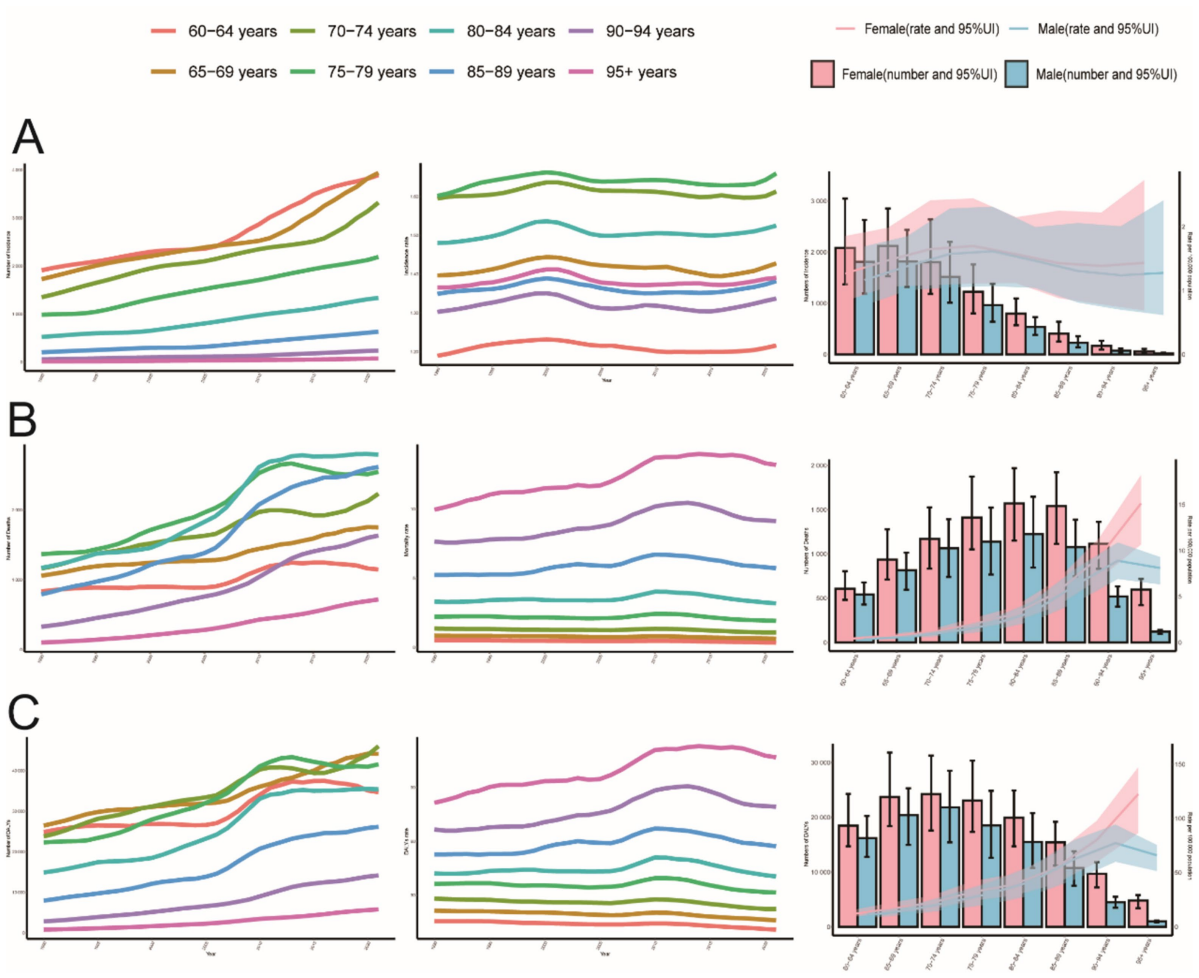
Trends in PAH incidence, mortality, and DALYs in older adults by age and sex, 1990–2021. **(A)** Incidence, cases and rates. **(B)** Mortality cases and rates. **(C)** Number of DALYs cases and rates.

**Figure 3:**
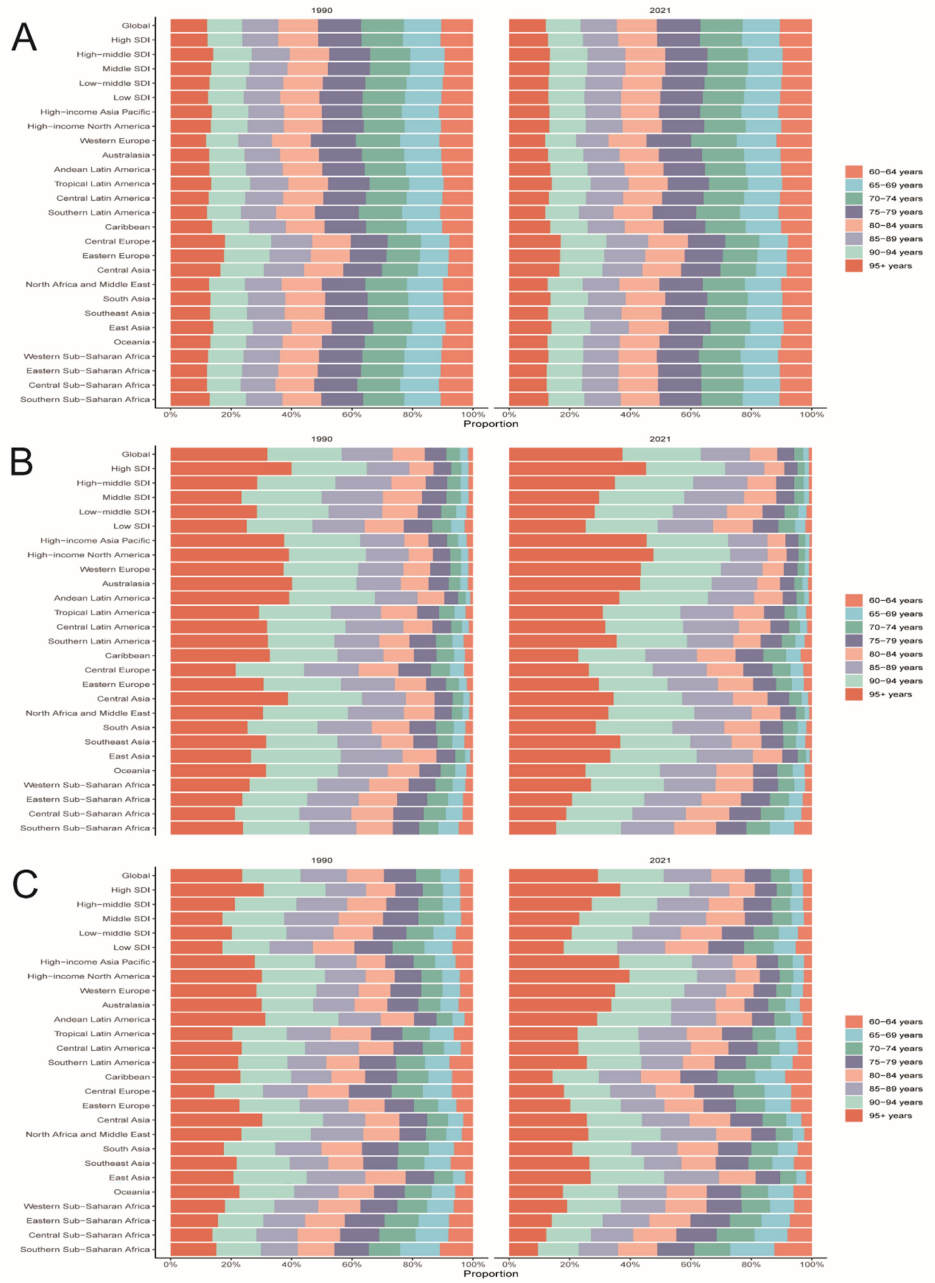
Age-specific percentages of PAH incidence, mortality, and DALYs in older age in 1990 and 2021. **(A)** incidence. **(B)** Death. **(C)** DALYs.

The global age-standardized incidence of PAH among adults aged ≥60 years increased from 1990 to 2021, with the steepest rise occurring between 1990 and 2000 (APC 34%, *p* < 0.05; Figure 1A) and a transient dip between 2015 and 2018. Globally, incident cases rose by 130%, while the EAPC was −0.01 (Table 1). The 75–79-year-old age group consistently had the highest incidence rates, and the 95+ year group had the lowest (Figures 2A, 3A). Incidence was higher in women across all age strata, with a 12% excess in the 95+ year-old group (Figure 2A).

**Table 1:** Global and regional incidence of PAH in older adults from 1990–2021.

Country	1990		2021		1990–2021
	Incidence cases	Incidence rate	Incidence cases	Incidence rate	EAPC
Global	6771.01 (4577.81, 9656.55)	1.40 (0.94, 2.01)	15622.29 (10546.44, 22296.65)	1.43 (0.97, 2.05)	−0.01 (−0.05, 0.02)
High SDI	1563.60 (1048.44, 2234.78)	1.08 (0.72, 1.55)	2907.46 (1934.65, 4201.55)	1.06 (0.71, 1.52)	−0.11 (−0.15, −0.07)
High-middle SDI	1576.11 (1059.56, 2267.55)	1.27 (0.85, 1.84)	3527.49 (2371.33, 5046.98)	1.37 (0.92, 1.97)	0.05 (−0.06, 0.16)
Middle SDI	1891.36 (1278.66, 2690.39)	1.61 (1.08, 2.30)	5194.15 (3517.43, 7401.04)	1.58 (1.06, 2.26)	−0.07 (−0.12, −0.02)
Low-middle SDI	1185.91 (810.66, 1681.03)	1.73 (1.18, 2.47)	2867.72 (1952.35, 4067.13)	1.69 (1.15, 2.40)	−0.20 (−0.24, −0.17)
Low SDI	546.81 (378.05, 771.61)	2.17 (1.49, 3.07)	1110.94 (762.38, 1573.35)	1.98 (1.35, 2.82)	−0.31 (−0.36, −0.27)
Andean Latin America	35.27 (23.68, 50.70)	1.50 (1.00, 2.16)	111.53 (74.85, 159.11)	1.55 (1.04, 2.21)	0.15 (0.05, 0.26)
Australasia	31.86 (20.91, 46.58)	1.02 (0.67, 1.50)	78.63 (51.53, 115.00)	1.12 (0.74, 1.63)	0.24 (0.19, 0.29)
Caribbean	41.47 (27.70, 59.66)	1.29 (0.86, 1.87)	91.44 (60.92, 131.82)	1.36 (0.91, 1.96)	0.25 (0.15, 0.36)
Central Asia	69.11 (45.93, 99.61)	1.26 (0.83, 1.81)	126.75 (85.16, 183.01)	1.35 (0.90, 1.95)	0.07 (−0.05, 0.19)
Central Europe	224.66 (150.39, 326.27)	1.18 (0.78, 1.73)	449.16 (300.16, 647.36)	1.48 (0.99, 2.14)	0.45 (0.29, 0.62)
Central Latin America	154.19 (104.06, 219.94)	1.62 (1.09, 2.32)	417.15 (277.53, 602.33)	1.35 (0.90, 1.95)	−0.31 (−0.52, −0.10)
Central Sub-Saharan Africa	54.55 (37.81, 76.89)	2.20 (1.51, 3.12)	134.16 (92.15, 189.92)	2.36 (1.61, 3.35)	0.42 (0.23, 0.62)
East Asia	1592.42 (1076.61, 2269.09)	1.58 (1.06, 2.26)	4270.66 (2893.46, 6072.28)	1.53 (1.03, 2.19)	−0.15 (−0.20, −0.11)
Eastern Europe	383.50 (251.40, 562.63)	1.07 (0.70, 1.59)	609.83 (404.69, 888.94)	1.28 (0.84, 1.86)	0.27 (−0.08, 0.63)
Eastern Sub-Saharan Africa	224.23 (155.73, 314.24)	2.69 (1.86, 3.80)	463.51 (322.60, 653.85)	2.55 (1.76, 3.61)	−0.02 (−0.10, 0.07)
High-income Asia Pacific	209.79 (137.09, 306.57)	0.83 (0.54, 1.21)	531.23 (346.11, 784.69)	0.90 (0.59, 1.32)	0.28 (0.23, 0.33)
High-income North America	356.91 (235.41, 519.75)	0.77 (0.51, 1.12)	797.39 (530.98, 1156.29)	0.90 (0.60, 1.30)	0.48 (0.38, 0.58)
North Africa and Middle East	322.00 (218.85, 457.22)	1.69 (1.14, 2.40)	803.06 (543.53, 1141.88)	1.56 (1.05, 2.22)	−0.46 (−0.67, −0.24)
Oceania	5.23 (3.59, 7.47)	1.66 (1.13, 2.38)	14.12 (9.62, 20.06)	1.81 (1.23, 2.57)	−0.03 (−0.15, 0.08)
South Asia	1045.72 (712.77, 1484.18)	1.66 (1.13, 2.37)	2921.26 (1987.36, 4141.43)	1.66 (1.12, 2.36)	−0.08 (−0.11, −0.05)
Southeast Asia	470.29 (319.05, 668.39)	1.65 (1.12, 2.36)	1342.35 (918.39, 1913.85)	1.72 (1.17, 2.46)	0.03 (−0.03, 0.10)
Southern Latin America	58.09 (38.21, 84.08)	0.98 (0.64, 1.43)	108.41 (71.26, 158.49)	0.96 (0.63, 1.41)	0.02 (−0.04, 0.08)
Southern Sub-Saharan Africa	67.85 (46.70, 95.63)	2.15 (1.48, 3.04)	135.91 (93.29, 192.20)	2.00 (1.36, 2.84)	−0.04 (−0.13, 0.06)
Tropical Latin America	154.77 (103.66, 220.38)	1.46 (0.97, 2.09)	456.35 (305.28, 652.73)	1.42 (0.95, 2.03)	−0.10 (−0.25, 0.06)
Western Europe	1035.83 (700.23, 1469.71)	1.36 (0.91, 1.93)	1388.05 (927.27, 2005.91)	1.18 (0.79, 1.69)	−0.53 (−0.62, −0.45)
Western Sub-Saharan Africa	233.27 (160.58, 329.69)	2.32 (1.59, 3.29)	371.33 (251.71, 532.65)	1.74 (1.17, 2.50)	−1.29 (−1.43, −1.14)

**Figure 4:**
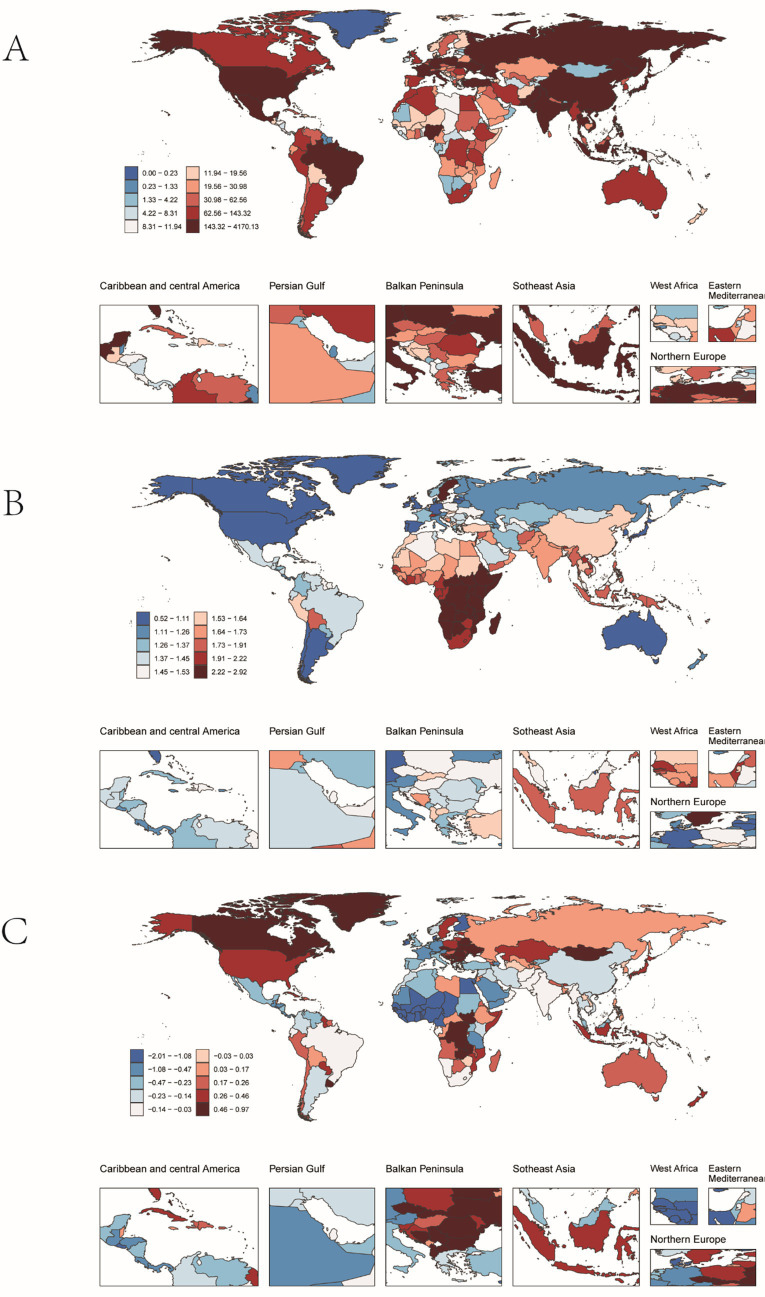
Incidence of pulmonary hypertension in older adults in 204 countries and territories. **(A)** The number of incidence cases. **(B)** Incidence. **(C)** Estimates Annual percent change (EAPC) in incidence.

In 2021, at the national level, China had the highest number of PAH cases worldwide (4,129 cases; 95% UI: 2797.25, 5872.71), while Zambia had the highest incidence rate (2.89 per 100,000 people; 95% UI: 2.00, 4.07) (Figures 4A,B). From 1990 to 2021, Slovakia showed the largest increase in incidence (EAPC = 0.96; 95% CI: 0.74, 1.19), whereas Burkina Faso showed the largest decrease (EAPC = −1.99; 95% CI: −2.31, −1.67) (Table 1 and Figure 4C). The global incidence of PAH was 1.43 (95% UI: 0.97, 2.05) per 100,000 people in 2021, higher than the incidence reported in 75 of 204 countries and lower than in 129 countries.

#### 3.1.2 Mortality

Globally, mortality from PAH remained stable until 2006, then increased during 2006–2011 (APC 3.4%, *p* < 0.05; Figure 1B), before levelling off. Total deaths rose by 130% over the study period, with an EAPC of 0.03 (Table 2). The 95+ year age group accounted for the highest proportion of deaths in both 1990 and 2021, while the 60–64-year group contributed less than 2% (Figures 2B, 3B). Female mortality exceeded male mortality across all age strata, with an 87% excess in the 95+ year group (Figure 2B).

**Table 2:** Global and regional deaths from PAH in older adults from 1990–2021.

Country	1990		2021		1990–2021
	Death cases	Death rate	Death cases	Death rate	EAPC
Global	6812.93 (5305.00, 8473.04)	1.64 (1.27, 2.04)	15443.42 (11746.25, 18388.02)	1.51 (1.15, 1.80)	0.03 (−0.16, 0.22)
High SDI	1738.27 (1517.21, 1937.64)	1.24 (1.07, 1.39)	3926.44 (3205.64, 4383.69)	1.22 (1.02, 1.36)	0.08 (−0.12, 0.29)
High-middle SDI	1753.71 (1449.36, 2223.61)	1.67 (1.36, 2.14)	3495.62 (2792.23, 4333.42)	1.44 (1.14, 1.78)	−0.22 (−0.48, 0.04)
Middle SDI	2241.76 (1582.82, 3045.00)	2.44 (1.71, 3.36)	5594.51 (3502.87, 6917.99)	2.00 (1.25, 2.47)	−0.17 (−0.47, 0.14)
Low-middle SDI	788.46 (398.09, 1279.76)	1.40 (0.69, 2.34)	1821.05 (1114.90, 2955.70)	1.26 (0.75, 2.11)	−0.15 (−0.23, −0.06)
Low SDI	284.59 (99.80, 517.29)	1.37 (0.46, 2.59)	596.59 (278.21, 1001.40)	1.29 (0.59, 2.20)	0.13 (−0.04, 0.30)
East Asia	2461.52 (1758.18, 3486.39)	3.57 (2.51, 5.17)	6362.16 (4144.16, 8049.38)	2.72 (1.78, 3.44)	−0.21 (−0.62, 0.20)
Southeast Asia	139.15 (68.88, 541.49)	0.57 (0.26, 2.46)	327.92 (192.10, 1212.02)	0.49 (0.27, 1.98)	−0.41 (−0.50, −0.32)
Oceania	2.89 (1.57, 6.52)	1.22 (0.66, 3.02)	5.81 (3.27, 14.05)	0.94 (0.52, 2.39)	−0.92 (−0.99, −0.86)
Central Asia	83.07 (57.40, 105.22)	1.70 (1.16, 2.17)	156.92 (127.23, 186.84)	2.01 (1.63, 2.39)	0.99 (0.70, 1.28)
Central Europe	223.91 (188.85, 258.81)	1.25 (1.05, 1.46)	346.04 (300.77, 390.78)	1.13 (0.98, 1.27)	−0.43 (−0.72, −0.14)
Eastern Europe	314.27 (284.53, 354.48)	0.99 (0.89, 1.12)	191.98 (172.82, 209.19)	0.41 (0.37, 0.45)	−3.49 (−3.88, −3.10)
High-income Asia Pacific	192.30 (171.55, 213.93)	0.84 (0.74, 0.94)	902.64 (678.88, 1061.44)	1.08 (0.85, 1.26)	0.90 (0.73, 1.07)
Australasia	29.05 (22.42, 39.10)	0.99 (0.76, 1.34)	44.27 (34.33, 54.29)	0.57 (0.44, 0.70)	−1.91 (−2.33, −1.48)
Western Europe	901.04 (768.78, 1035.49)	1.18 (1.00, 1.35)	1570.43 (1288.15, 1772.48)	1.05 (0.88, 1.18)	−0.15 (−0.72, 0.43)
Southern Latin America	68.30 (55.45, 83.14)	1.24 (1.00, 1.51)	93.45 (77.90, 110.07)	0.81 (0.68, 0.96)	−1.16 (−1.43, −0.88)
High-income North America	763.67 (649.36, 862.97)	1.62 (1.38, 1.84)	1581.04 (1303.12, 1758.14)	1.68 (1.39, 1.86)	−0.03 (−0.19, 0.14)
Caribbean	33.36 (27.31, 40.83)	1.11 (0.91, 1.37)	32.04 (24.95, 48.77)	0.47 (0.37, 0.72)	−3.56 (−3.94, −3.19)
Andean Latin America	25.01 (17.91, 33.47)	1.20 (0.85, 1.60)	52.38 (38.34, 75.29)	0.76 (0.55, 1.09)	−1.33 (−1.61, −1.05)
Central Latin America	63.67 (51.86, 74.75)	0.77 (0.63, 0.91)	123.39 (105.61, 143.87)	0.42 (0.36, 0.49)	−2.39 (−2.79, −1.99)
Tropical Latin America	144.47 (130.37, 157.30)	1.57 (1.40, 1.72)	484.64 (414.49, 537.04)	1.57 (1.34, 1.75)	0.02 (−0.55, 0.58)
North Africa and Middle East	426.68 (257.11, 658.29)	2.92 (1.70, 4.57)	925.95 (607.17, 1176.22)	2.28 (1.49, 2.90)	−0.34 (−0.50, −0.18)
South Asia	731.43 (311.08, 1358.30)	1.41 (0.56, 2.75)	1919.51 (1025.03, 3600.80)	1.28 (0.66, 2.50)	−0.07 (−0.21, 0.08)
Central Sub-Saharan Africa	21.93 (7.27, 49.98)	1.11 (0.35, 2.67)	45.84 (16.65, 97.91)	0.97 (0.35, 2.12)	−0.34 (−0.40, −0.29)
Eastern Sub-Saharan Africa	77.57 (22.07, 179.71)	1.11 (0.31, 2.71)	117.62 (40.96, 242.66)	0.76 (0.27, 1.60)	−1.31 (−1.34, −1.27)
Southern Sub-Saharan Africa	13.17 (7.61, 23.18)	0.45 (0.25, 0.83)	26.93 (18.41, 36.93)	0.42 (0.29, 0.61)	−0.05 (−0.28, 0.19)
Western Sub-Saharan Africa	96.49 (23.23, 234.09)	1.13 (0.27, 2.86)	132.48 (41.95, 268.86)	0.75 (0.24, 1.55)	−1.49 (−1.60, −1.38)

**Figure 5:**
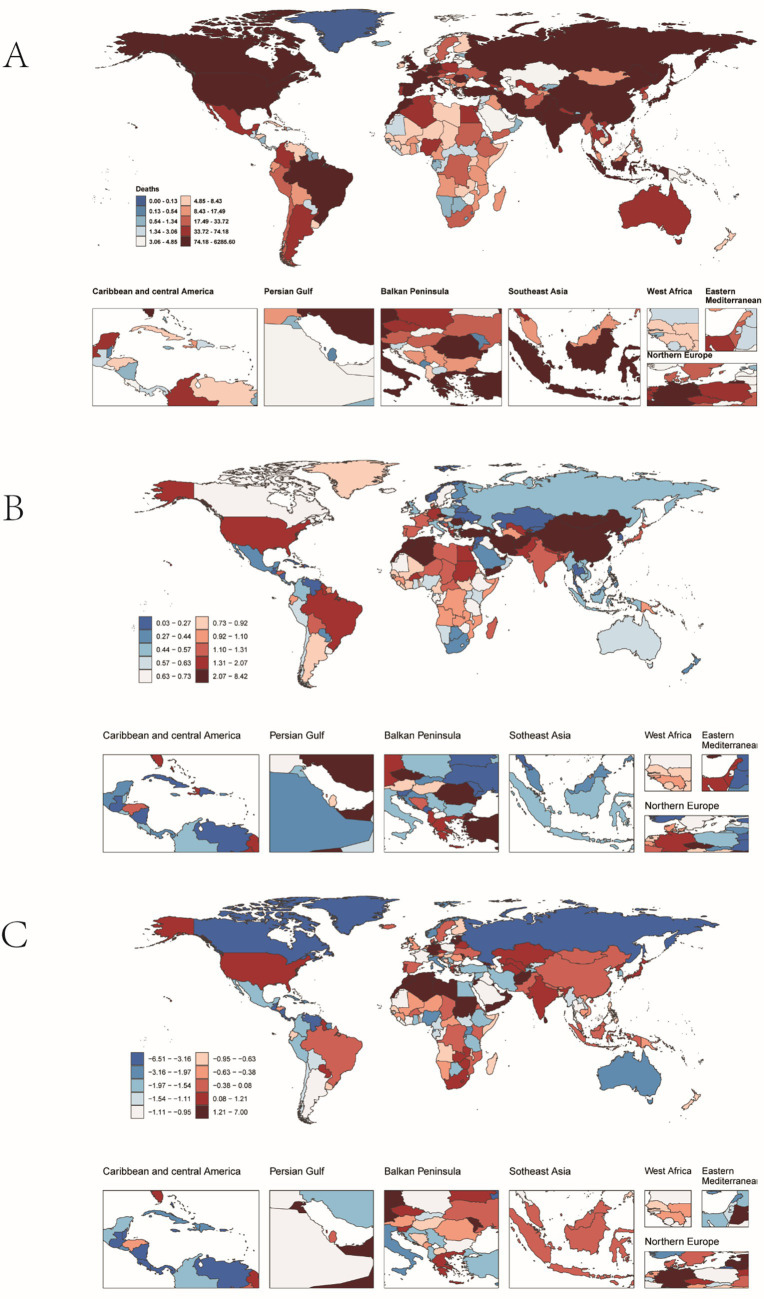
Mortality from pulmonary hypertension in older adults in 204 countries and territories. **(A)** Number of deaths. **(B)** Mortality. **(C)** Estimates annual percentage change (EAPC) in mortality.

In 2021, China had the highest number of PAH-related deaths among the regions considered (6223.37 cases; 95% UI: 4016.90, 7896.42), while the Democratic People’s Republic of Korea had the highest mortality rate (2.18 per 100,000 people; 95% UI: 1.19, 4.01) (Figures 5A,B). From 1990 to 2021, Latvia saw the largest increase in mortality (EAPC = 6.93; 95% CI: 4.63, 9.28), whereas Puerto Rico saw the largest decrease (EAPC = −6.45; 95% CI: −6.83, −6.06) (Figure 5C). The global mortality rate of PAH was 1.51 (95% UI: 1.15, 1.80) per 100,000 people in 2021, higher than the rate in 35 of 204 countries and lower than in 169 countries.

#### 3.1.3 DALYs

Globally, DALYs due to PAH remained stable until 2006, peaked in 2010, and rose most rapidly during 2006–2011 (APC 3.0%, *p* < 0.05; Figure 1C). DALYs increased by 100% over the study period, while the age-standardized rate showed an EAPC of −0.23 (Table 3). The 95+ year age group consistently had the highest DALY rates, and women experienced an 86% higher rate than men in the oldest stratum (Figure 2C).

**Table 3:** Global and regional DALYs of PAH in older adults, 1990–2021.

Country	1990		2021		1990–2021
	DALYs cases	DALYs rate	DALYs cases	DALYs rate	EAPC
Global	124028.51 (97637.33, 153493.70)	27.36 (21.47, 33.86)	248063.85 (190570.38, 293817.80)	23.54 (18.07, 27.89)	−0.23 (−0.41, −0.05)
High SDI	29626.16 (26525.96, 32706.75)	20.71 (18.45, 22.90)	56591.85 (48378.40, 62438.28)	18.96 (16.45, 20.82)	−0.23 (−0.42, −0.03)
High-middle SDI	31719.85 (26622.29, 39438.95)	27.39 (22.79, 34.41)	55513.71 (44966.37, 68274.30)	22.22 (17.97, 27.35)	−0.45 (−0.69, −0.21)
Middle SDI	41266.83 (29632.21, 55386.36)	39.33 (27.95, 53.28)	90961.64 (58423.25, 112093.81)	30.13 (19.21, 37.14)	−0.39 (−0.68, −0.09)
Low-middle SDI	15409.72 (8141.76, 24098.80)	24.16 (12.44, 38.73)	33288.93 (21467.11, 51340.14)	21.03 (13.24, 33.27)	−0.31 (−0.39, −0.23)
Low SDI	5889.24 (2200.07, 10368.45)	24.70 (8.89, 44.68)	11546.58 (5664.20, 18960.34)	22.23 (10.64, 36.94)	−0.11 (−0.23, 0.01)
East Asia	44333.12 (32125.44, 61948.92)	53.50 (38.19, 76.13)	99304.79 (64804.35, 125791.27)	39.18 (25.62, 49.61)	−0.31 (−0.72, 0.10)
Southeast Asia	2882.75 (1566.07, 9670.13)	10.43 (5.41, 38.43)	6551.14 (4185.63, 20627.43)	8.79 (5.43, 30.03)	−0.50 (−0.58, −0.41)
Oceania	61.98 (34.16, 132.71)	21.48 (11.85, 49.32)	119.19 (68.91, 274.74)	16.47 (9.44, 39.49)	−0.91 (−0.99, −0.84)
Central Asia	1412.49 (1017.74, 1751.91)	27.22 (19.39, 33.95)	2757.09 (2232.03, 3291.94)	32.21 (26.17, 38.23)	0.93 (0.63, 1.24)
Central Europe	4297.39 (3661.20, 4928.97)	22.55 (19.14, 26.00)	6119.65 (5381.25, 6860.29)	20.15 (17.72, 22.59)	−0.48 (−0.77, −0.19)
Eastern Europe	5933.53 (5398.68, 6777.26)	17.17 (15.55, 19.53)	3716.07 (3371.54, 4079.12)	7.78 (7.05, 8.54)	−3.20 (−3.56, −2.84)
High-income Asia Pacific	3689.03 (3365.84, 4057.01)	15.09 (13.64, 16.68)	11956.07 (9546.20, 13838.99)	16.63 (13.76, 19.07)	0.34 (0.20, 0.49)
Australasia	520.33 (409.67, 691.84)	17.12 (13.40, 22.76)	703.77 (566.04, 848.36)	9.56 (7.73, 11.50)	−2.01 (−2.42, −1.61)
Western Europe	15276.61 (13290.87, 17316.82)	19.84 (17.21, 22.50)	22442.90 (19128.99, 25066.40)	16.64 (14.43, 18.48)	−0.45 (−0.98, 0.08)
Southern Latin America	1320.22 (1078.02, 1596.51)	22.77 (18.56, 27.58)	1662.33 (1406.51, 1936.68)	14.65 (12.40, 17.06)	−1.29 (−1.55, −1.03)
High-income North America	12585.22 (11039.25, 14067.60)	26.85 (23.53, 30.03)	23123.11 (19890.99, 25323.12)	25.29 (21.87, 27.64)	−0.40 (−0.57, −0.23)
Caribbean	634.19 (520.48, 770.44)	20.16 (16.54, 24.58)	619.10 (489.88, 881.50)	9.20 (7.28, 13.09)	−3.30 (−3.66, −2.94)
Andean Latin America	409.89 (299.20, 540.48)	18.51 (13.47, 24.46)	840.93 (626.00, 1183.80)	11.95 (8.89, 16.83)	−1.32 (−1.56, −1.08)
Central Latin America	1146.50 (937.76, 1327.87)	12.84 (10.50, 14.93)	2266.67 (1973.81, 2634.05)	7.53 (6.56, 8.75)	−2.19 (−2.56, −1.82)
Tropical Latin America	2797.32 (2550.66, 3039.12)	27.52 (24.90, 29.98)	8388.49 (7379.21, 9191.95)	26.57 (23.30, 29.14)	−0.21 (−0.76, 0.35)
North Africa and Middle East	7716.65 (4814.49, 11686.92)	45.88 (27.91, 70.44)	15294.66 (10208.43, 19459.03)	33.97 (22.51, 43.15)	−0.64 (−0.77, −0.52)
South Asia	14647.00 (6702.87, 25772.97)	24.67 (10.75, 45.39)	35474.44 (20183.69, 62196.79)	21.46 (11.84, 39.06)	−0.29 (−0.39, −0.18)
Central Sub-Saharan Africa	491.49 (176.74, 1075.85)	20.96 (7.33, 47.69)	986.98 (376.85, 2037.59)	18.08 (6.88, 38.18)	−0.38 (−0.45, −0.32)
Eastern Sub-Saharan Africa	1652.18 (510.32, 3662.41)	20.66 (6.28, 47.53)	2427.22 (904.59, 4785.47)	13.97 (5.20, 28.06)	−1.39 (−1.43, −1.35)
Southern Sub-Saharan Africa	289.12 (179.28, 470.47)	9.14 (5.60, 15.33)	615.70 (441.11, 792.81)	8.92 (6.35, 11.82)	0.05 (−0.20, 0.31)
Western Sub-Saharan Africa	1931.48 (513.09, 4413.98)	20.20 (5.28, 47.77)	2693.54 (941.41, 5184.78)	13.53 (4.71, 26.48)	−1.45 (−1.56, −1.35)

**Figure 6:**
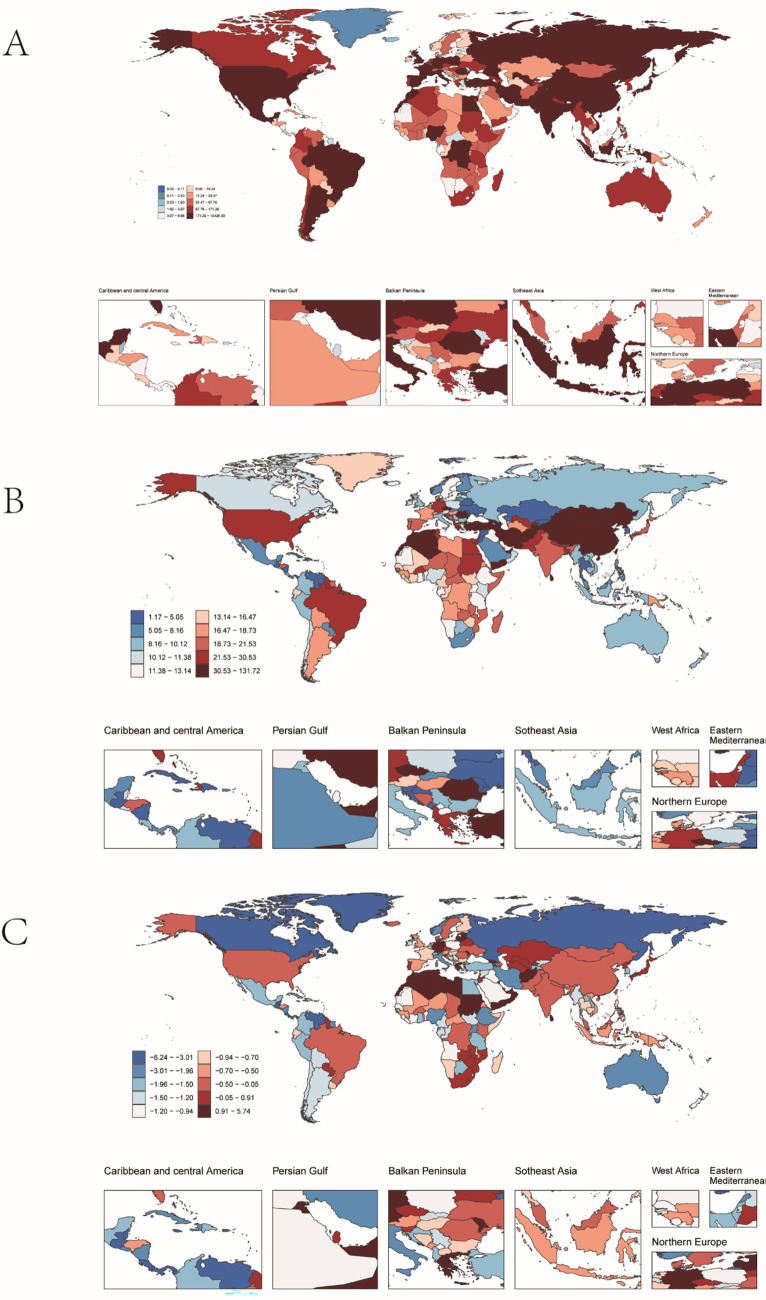
PAH DALYs in older adults in 204 countries and territories. **(A)** The number of DALYs. **(B)** DALYs rate. **(C)** Estimates annual percentage change (EAPC) in DALYs.

In 2021, China had the highest number of DALYs for PAH globally (19,236; 95% UI: 12257.42, 24197.57), while Mongolia had the highest DALY rate (130.41 per 100,000 people; 95% UI: 70.47, 187.59) (Figures 6A,B). From 1990 to 2021, Georgia saw the greatest increase in DALYs (EAPC = 5.68; 95% CI: 4.81, 6.56), whereas Puerto Rico saw the greatest decrease (EAPC = −6.18; 95% CI: −6.57, −5.79) (Figure 6C). The global DALY rate of PAH was 23.54 (95% UI: 18.07, 27.89) per 100,000 people in 2021, higher than the rate in 40 of 204 countries and lower than in 166 countries.

### 3.2 PAH in older people: regional trends by SDI

From 1990 to 2021, the incidence, mortality, and DALYs of PAH in individuals aged over 60 years across different SDI regions generally showed a declining trend, though with variations in the magnitude of change. In 2021, the middle-SDI region had the highest incidence rate (95% UI: 1.06, 2.26), while the high-SDI region had the lowest incidence rates in both 1990 (1.08, 95% UI: 0.72, 1.55) and 2021 (1.06, 95% UI: 0.71, 1.52).

For mortality, the middle-SDI region also had the highest death rate in 2021 (2.00; 95% UI: 1.25, 2.47), with the high-SDI region having the lowest death rates in both 1990 (1.24, 95% UI: 1.07–1.39) and 2021 (1.22, 95% UI: 1.02, 1.36).

Regarding DALYs, the middle-SDI region had the highest DALY rate in 2021 (30.13; 95% UI: 19.21, 37.14), whereas the high-SDI region had the lowest DALY rates in both 1990 (20.71; 95% UI: 18.45, 22.90) and 2021 (18.96; 95% UI: 16.45, 20.82).

**Figure 7:**
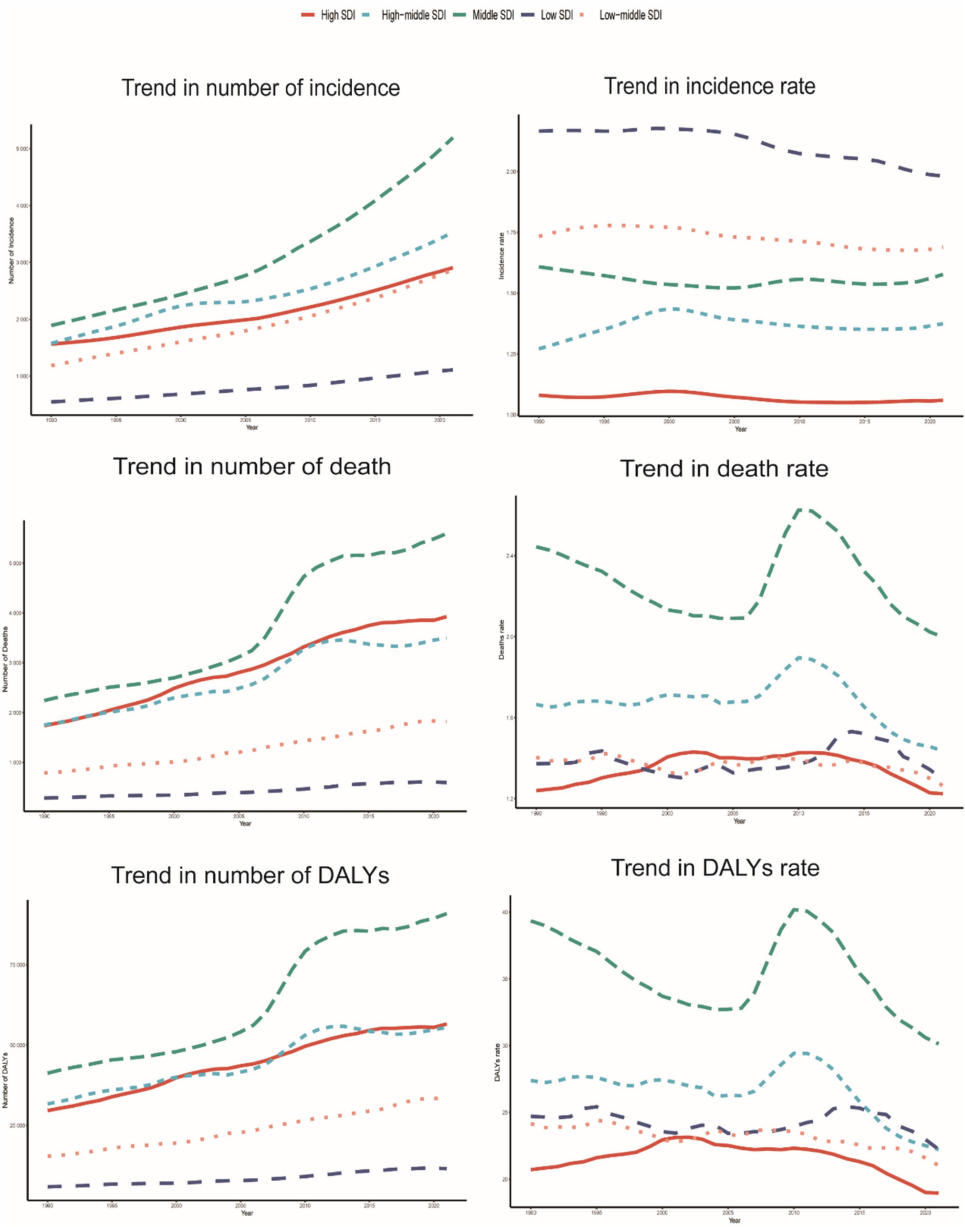
Epidemiological trends in the incidence, mortality, and DALYs rates of PAH in five older adults Sociodemographic Index (SDI) region from 1990 to 2021.

**Figure 8:**
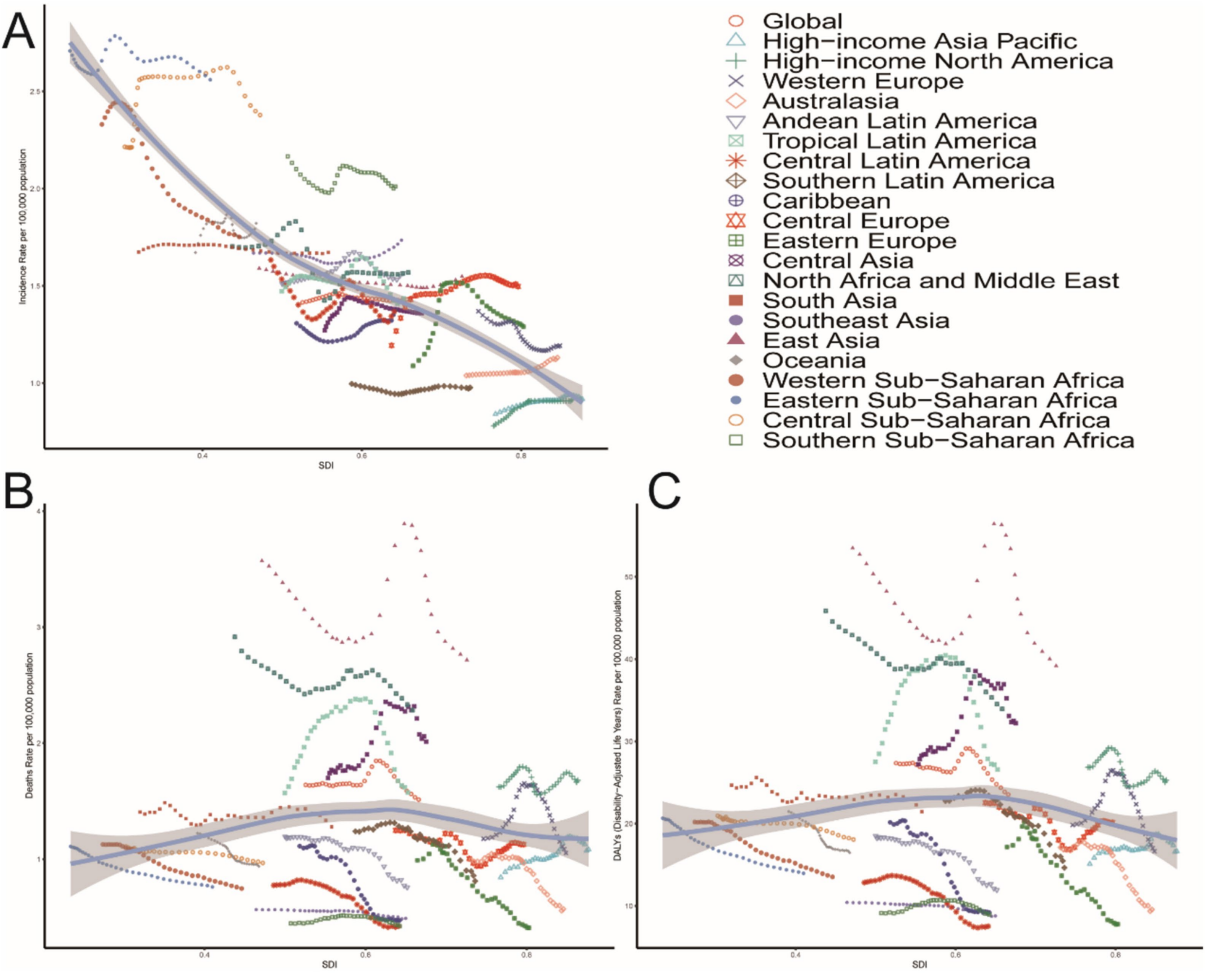
Association between the incidence, mortality, and DALYs rates of PAH in older adults and the regional incidence of PAH, mortality, and DALYs. Sociodemographic Index (SDI), 1990–2021. **(A)** Incidence rate. **(B)** Mortality rate. **(C)** DALYs rates.

However, the low-SDI region experienced the most significant decrease in incidence (EAPC = −0.31; 95% CI: −0.36, −0.27), while the high-SDI region showed the most substantial decline in the DALY rate (EAPC = −0.23; 95% CI: −0.42, −0.03) (Table 1 and Figures 7, 8).

### 3.3 PAH in older people: projections for 2050

#### 3.3.1 Incidence

**Figure 9:**
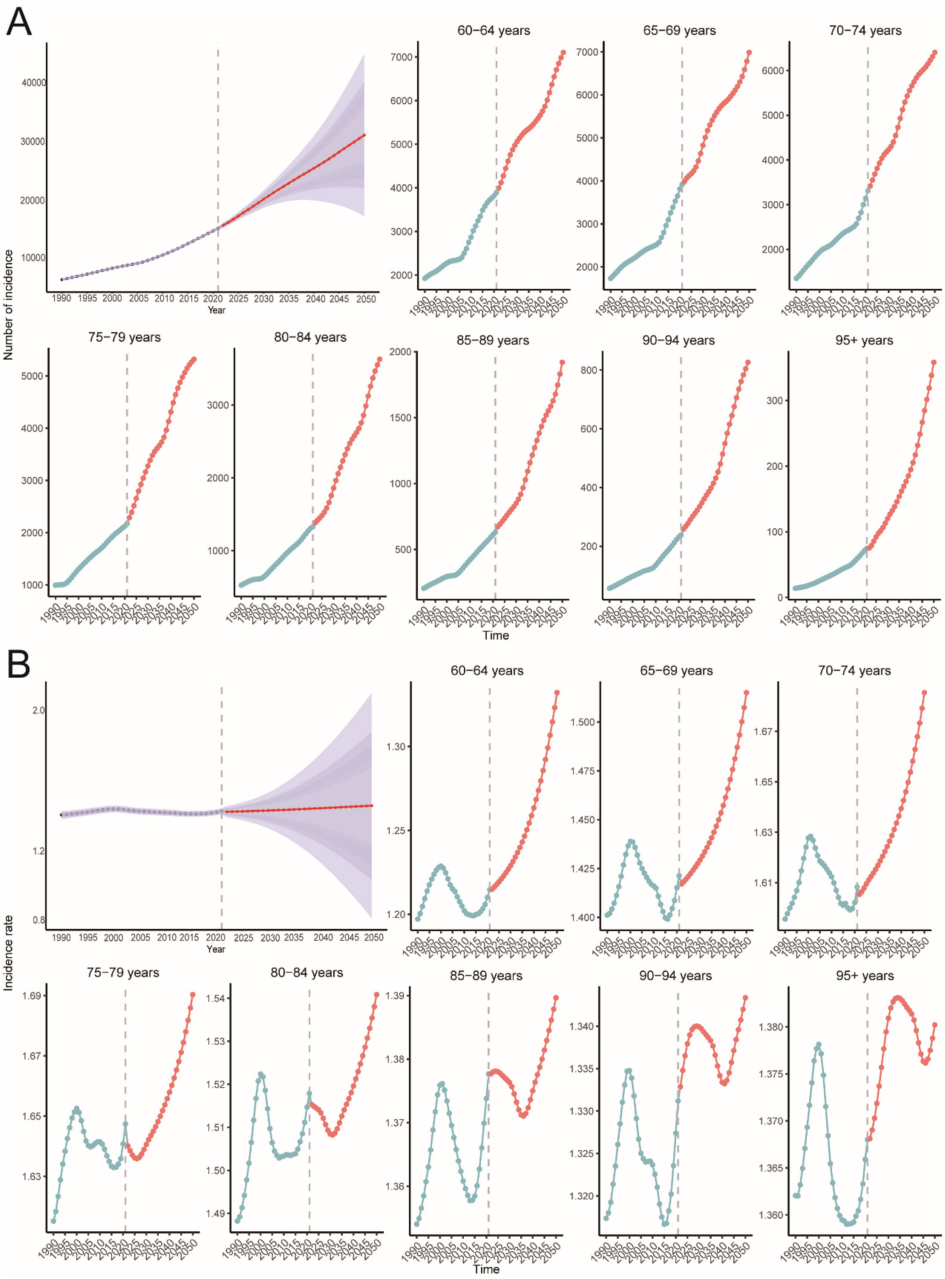
Predict the total number of elderly PAH cases and the incidence rates for different age groups in 2050. **(A)** Number of incidence. **(B)** Incidence rate.

From 1990 to 2050, the global incidence of PAH in individuals aged 60 years and older is projected to increase significantly. By 2050, the global number of PAH cases in this age group is expected to reach 31,383 (95% CI: 17604.40, 45162.56) (Figure 9A), with an incidence rate of 1.46 (95% CI: 0.82, 2.10) per 100,000 people (Figure 9B). The highest number of new cases is projected to be in the 85–89-year age group, with 7,105 cases (95% CI: 1888.57, 12320.77) (Figure 9A). The highest incidence rate is expected to be in the 75–79-year age group, at 1.69 (95% CI: 1.21, 2.17) per 100,000 people (Figure 9B).

#### 3.3.2 Mortality

**Figure 10:**
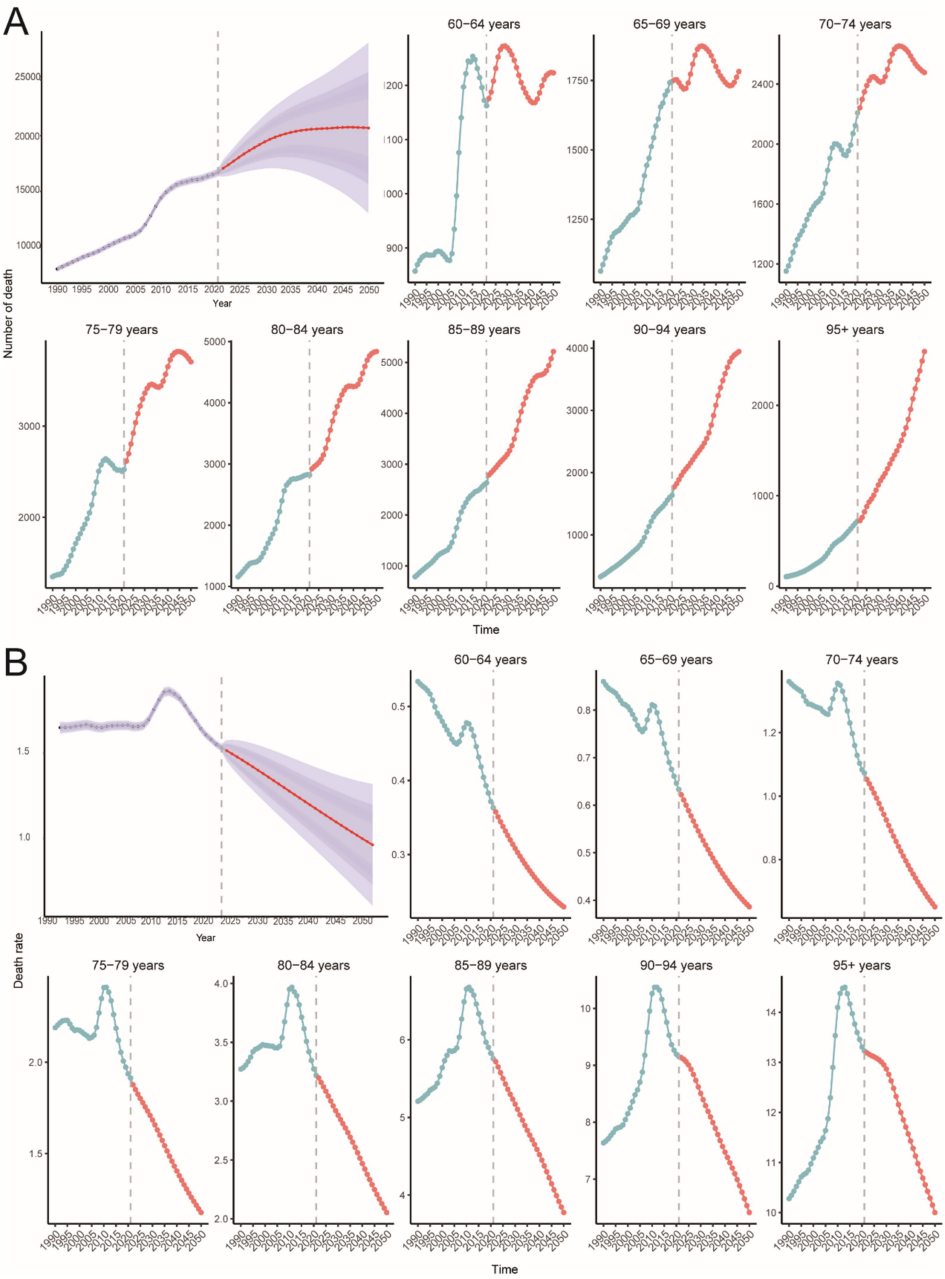
Predict the total number of elderly PAH death cases and the death rates for different age groups in 2050. **(A)** Number of death. **(B)** Death rate.

The global number of PAH-related deaths in individuals aged 60 years and older is projected to reach 20,687 (95% CI: 13016.47, 28358.44) by 2050 (Figure 10A), with a death rate of 0.96 (95% CI: 0.60, 1.32) per 100,000 people (Figure 10B). The highest number of deaths is predicted to be in the 85–89-year age group, with 5,214 deaths (95% CI: 3738.05, 6689.92) (Figure 10A). The highest death rate is expected to be in the 95+ age group, at 14.50 (95% CI: 13.95, 15.05) per 100,000 people (Figure 10B).

#### 3.3.3 DALYs

**Figure 11:**
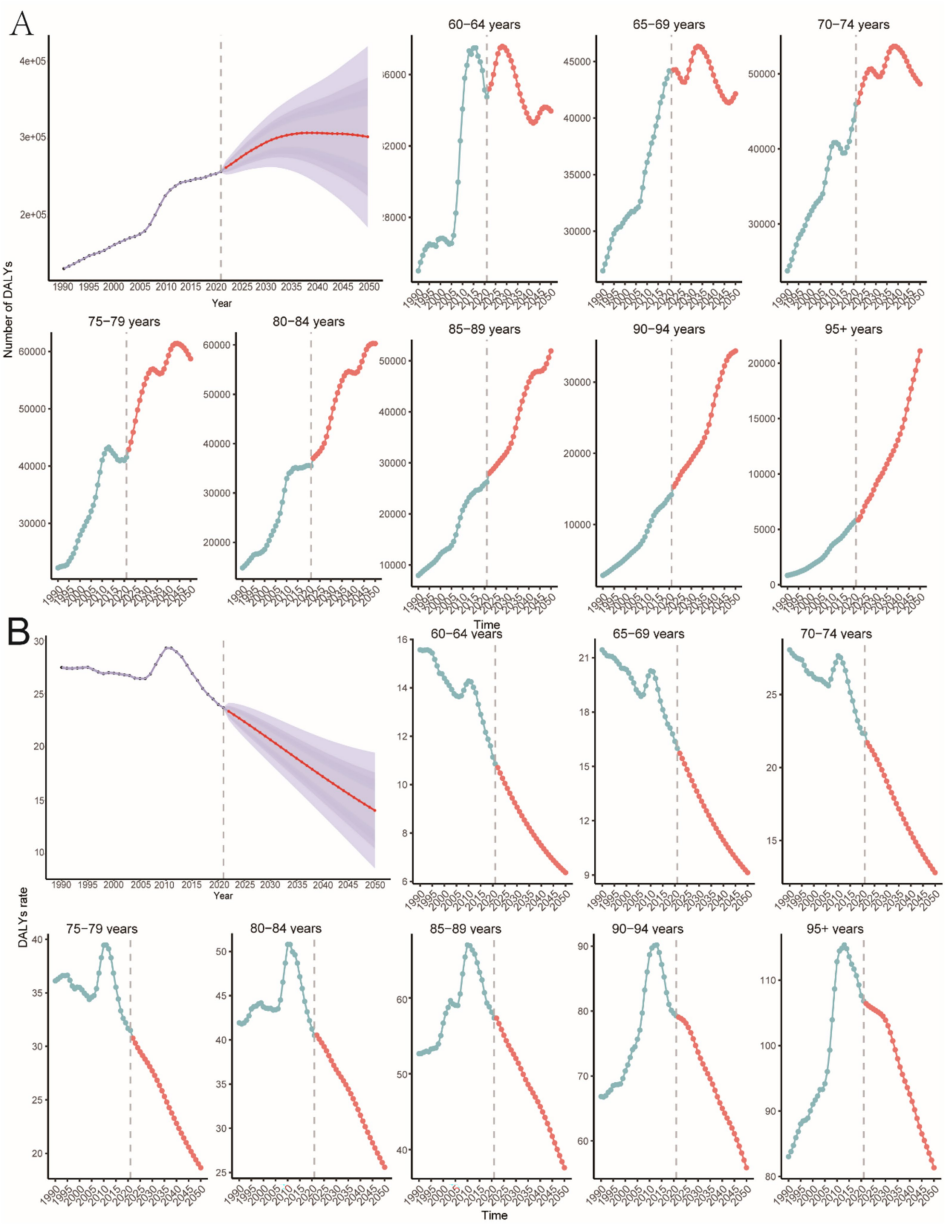
Predicting the total number of DALYs in older PAHs and the rate of DALYs in different age groups in 2050. **(A)** Number of DALYs. **(B)** Rate of DALYs.

The global number of PAH-related DALYs in individuals aged 60 years and older is projected to reach 302,760 (95% CI: 186486.36, 419033.63) by 2050 (Figure 11A), with a DALY rate of 14.08 (95% CI: 8.67, 19.48) per 100,000 people (Figure 11B). The highest number of DALYs is predicted to be in the 75–79-year age group, with 61,411 DALYs (95% CI: 46693.37, 76128.41) (Figure 11A). The highest DALY rate is expected to be in the 95+ age group, at 115.38 (95% CI: 112.83, 117.93) per 100,000 people (Figure 11B).

## 4 Discussion

This study provides a comprehensive analysis of the global burden of PAH among individuals aged 60 and older from 1990 to 2021 and projects trends through 2050. Key findings indicate that during this period, global PAH incidence, mortality, and DALYs increased by 130, 130, and 100%, respectively. By 2021, there were 15,622 new cases (incidence rate: 1.43 per 100,000 people; 95% UI: 0.97, 2.05), 15,443 deaths (mortality rate: 1.51 per 100,000 people; 95% UI: 1.15, 1.80), and 248,064 DALYs (DALY rate: 23.54 per 100,000 people; 95% UI: 18.07, 27.89) globally. The age-standardized incidence rate rose from 1.447 to 1.66 per 100,000 population. The disease burden is unevenly distributed globally, with regions of medium SDI bearing the heaviest burden. China reported the highest number of cases and deaths, while Zambia had the highest incidence rate. Projections using the BAPC model indicate that the burden of PAH in the elderly population will continue to rise through 2050: the number of new cases is expected to reach 31,383 (95% CI: 17604.40, 45162.56) with an incidence rate of 1.46 per 100,000, approximately 20,687 deaths, and 302,760 DALYs. These findings underscore PAH as a significant and growing public health challenge within an increasingly aging population.

PAH is a serious disease that profoundly impacts the quality of life in the elderly and imposes a heavy burden on healthcare systems (16). The stable or slightly declining age-standardized rates of incidence and DALYs (EAPC = −0.01 for incidence, −0.23 for DALYs) indicate that the increase in absolute burden is primarily driven by population aging, while per capita risk is modulated by other factors. Lower-than-expected mortality can be attributed to significant advances in therapeutic strategies over the past two decades, including the development of targeted therapies, effective risk management, combination therapies, and improved diagnostic techniques, which collectively have enhanced survival rates among PAH patients (17). The transient decline in incidence observed between 2015 and 2018 may reflect temporary shifts in diagnostic or coding practices, warranting further investigation.

The burden of PAH varies significantly across socio-demographic regions, with the highest burden observed in areas with moderate SDI in 2021. This aligns with the complex interplay between an aging population and potentially lagging healthcare infrastructure. Despite population aging, the overall incidence trend shows a slight decline, which may be attributable to significant advances in early detection, improved screening programs, and heightened clinical awareness. The adoption of new risk assessment tools has facilitated earlier diagnosis, enabling more effective therapeutic interventions (18). Furthermore, the use of multimodal therapies has contributed to reducing mortality associated with PAH (19, 20). In contrast, high SDI regions consistently exhibit the lowest incidence rates, likely due to better integrated care and access to advanced treatments. The declining trend in low SDI regions may reflect improved public health interventions, although the possibility of undiagnosed cases and data limitations cannot be ruled out in these areas. This is evidenced by the substantial diagnostic gap between HICs and LICs within the GBD database.

Variations in the disease burden of PAH across different SDI regions may be influenced by specific national health policies, healthcare accessibility, and the pace of population aging (21). Medium-SDI regions, experiencing rapid population aging, may have healthcare systems that are not yet fully equipped to manage complex chronic diseases like PAH (22). This can lead to high diagnosis rates but limited treatment capacity, thereby increasing the overall burden. In contrast, high SDI regions typically have stronger primary care systems and specialist referral networks, which facilitate early detection and management of PAH, thus controlling its progression (8). The declining trend in low SDI regions may reflect inadequate diagnostic capabilities. A PAH diagnosis requires meeting the “hemodynamic definition of pulmonary hypertension (PH),” excluding other causes of precapillary pulmonary hypertension, and satisfying clinical/classification criteria (23). Future research should integrate specific national health policies and healthcare resource allocation to better understand the structural drivers behind these SDI disparities.

At the national level, China reported the highest number of pulmonary arterial hypertension (PAH) cases and deaths, consistent with its large and aging population. Zambia had the highest incidence rate, potentially linked to specific environmental or genetic risk factors. The significant increase in incidence in Slovakia and the marked rise in mortality in Latvia highlight the need for further investigation into local risk factors and healthcare system performance.

Projections using the BAPC model indicate that the burden of PAH in the elderly population will continue to rise through 2050. The highest incidence is projected for the 75–79 age group, while the highest mortality is anticipated in the 95+ age cohort. Increased stiffness in the cardiac and pulmonary vascular systems among the elderly elevates the risk of developing PAH (24). This underscores the need for age-specific prevention and care strategies in the context of accelerating global population aging (25).

From a gender perspective, women exhibit higher incidence, mortality, and DALYs rates than men across most age groups, particularly among those aged 60 and older. Previous studies have shown that the Y chromosome exerts a protective effect in hypoxia-induced PAH, reducing right ventricular systolic pressure and pulmonary vascular remodelling (26). It has been hypothesized that genes such as SRY and UTY may confer protection against PAH by enhancing BMP signalling and reducing proinflammatory cytokines (27, 28). However, with advancing age, the gradual loss of Y chromosome fragments in male peripheral cells or reduced expression of Y chromosome protective genes may diminish this protective effect, contributing to increased disease incidence in older males (29, 30). Conversely, estradiol has demonstrated bidirectional effects in PAH (the estrogen paradox), which may also contribute to the observed gender differences (31). Additionally, women tend to be more sensitive to early symptoms, such as shortness of breath, and are more likely to seek medical care promptly, which facilitates earlier diagnosis (32). In contrast, elderly men often attribute such symptoms to aging or comorbidities, resulting in delayed medical consultations and underdiagnosis. This disparity is further amplified by unequal access to healthcare resources. In regions with a high to medium-SDI, women achieve higher diagnosis rates due to easier access to regular check-ups. In regions with a low-SDI, some women still receive diagnoses despite widespread diagnostic limitations, while male patients remain entirely undiagnosed due to their near-total avoidance of medical care. Therefore, addressing this issue requires biomedical interventions and the implementation of gender-sensitive strategies, such as proactive screening of high-risk males, to address all contributing factors comprehensively.

Using GBD 2021, we provide a systematic, global snapshot of PAH in adults aged greater than 60 years. Our findings show that the burden is highest in middle-SDI countries; China contributes the largest absolute number of cases, while Zambia records the highest age-standardized incidence; older women consistently face greater risk than men. These epidemiological insights offer immediate, location-specific targets, such as intensified screening in middle-SDI settings and sex-tailored management for elderly women. Additionally, our BAPC projections provide actionable foresight for health-system planning toward 2050, when one in six people worldwide will be over 65.

However, this study has several limitations. The GBD estimates we used can under- or overstate true rates due to weak death registration and patchy diagnostic capacity in many low- and middle-income countries. The GBD also lacks data on air pollution, smoking, drug use, or local access to care, limiting our ability to explain why rates rise or fall in specific places. The absence of individual-level information means we cannot adjust for genetic background, comorbidities, or treatment adherence, and the clinical meaning of the estimates is limited. These data issues directly affect our trend tests. The UI provided by GBD is designed for single-year comparisons, not for tracking changes over time. We used non-overlapping UI to judge differences, but this approach ignores the serial correlation in time series and may inflate false positives. Joinpoint results depend on user choices, such as minimum observations per segment and maximum number of joinpoints, which can alter conclusions about recent trends. The EAPC assumes a constant log-linear slope from 1990 to 2021, averaging out any plateaus, rebounds, or declines, which may misrepresent trends as “stable” when they are not. For the 2050 projections, the BAPC model assumes that age, period, and cohort effects observed in only six five-year periods will remain unchanged for the next 29 years. The coarse age groups (eight five-year bands above 60) and thirteen ten-year birth cohorts smooth out shocks such as war or drug-era fluctuations, likely overstating forecast precision. Finally, our curve fitting between SDI and PAH burden uses an ecological composite index tied to the same social variables that shape healthcare access; the association is descriptive and cannot be taken as causal.

## 5 Conclusion

This study highlights the substantial and growing burden of PAH among the global elderly population, with a disproportionate impact on female and middle-SDI countries. Projections indicate a marked escalation by 2050, although these estimates assume stable trends and should be viewed with caution. The findings underscore the urgent need for targeted public health strategies, including enhanced early detection, sex-specific management, and stronger data systems, to mitigate PAH’s impact in an aging world.

## Data Availability

Publicly available datasets were analyzed in this study. This data can be found here: https://vizhub.healthdata.org/gbd-results/.
