# Supplementary figures and images for: Global, regional, and national burden of pulmonary arterial hypertension in adults aged ≥60 years from 1990–2021: SDI-stratified trend analysis and projections to 2050
Source: Front Med (Lausanne). 2025 Nov 25;12:1595504. doi: 10.3389/fmed.2025.1595504 (PMC12685917; doi:10.3389/fmed.2025.1595504)

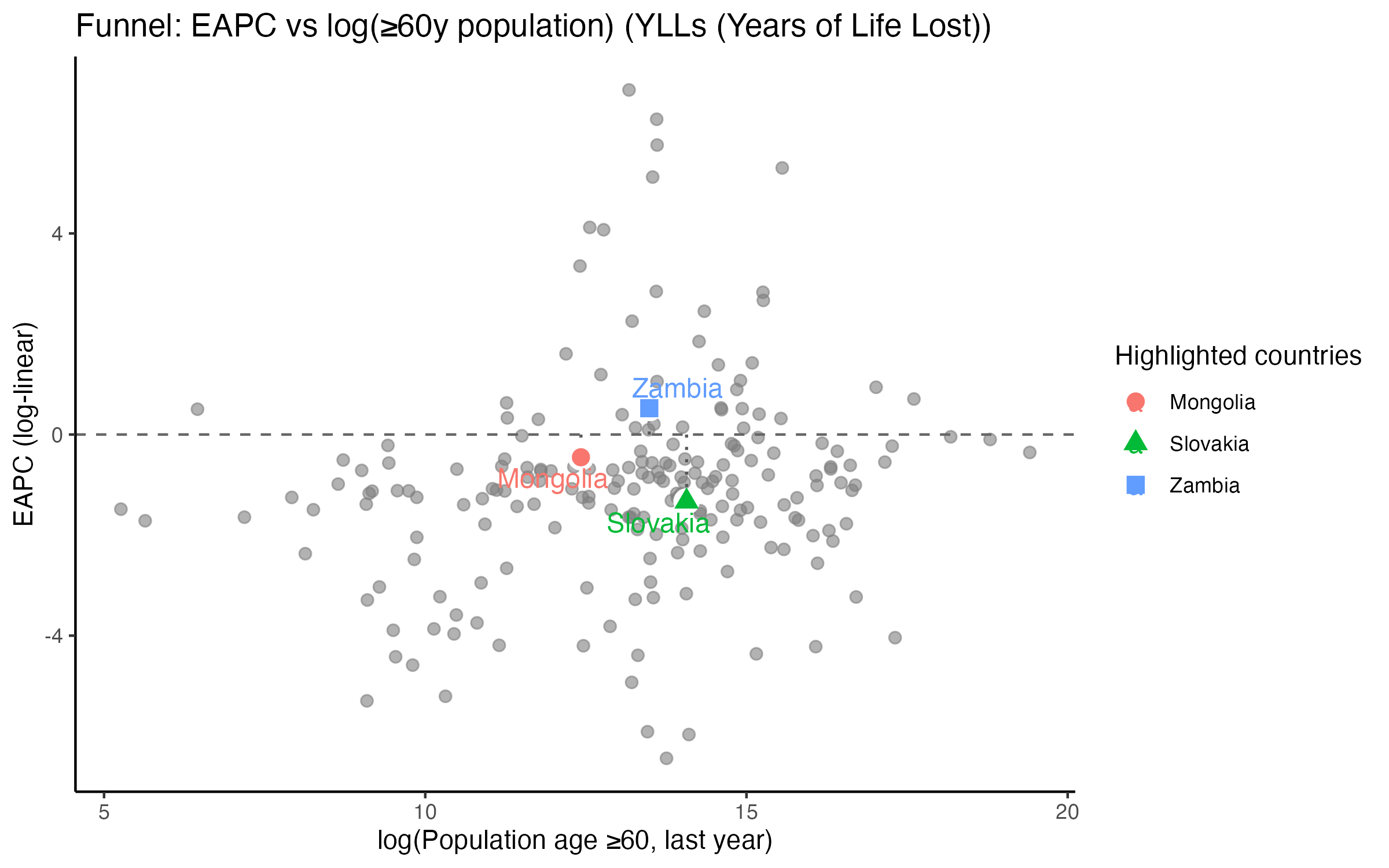

Supplement: Supplementary file 1 [file Data_Sheet_1.zip › funnel_YLLs (Years of Life Lost).png]

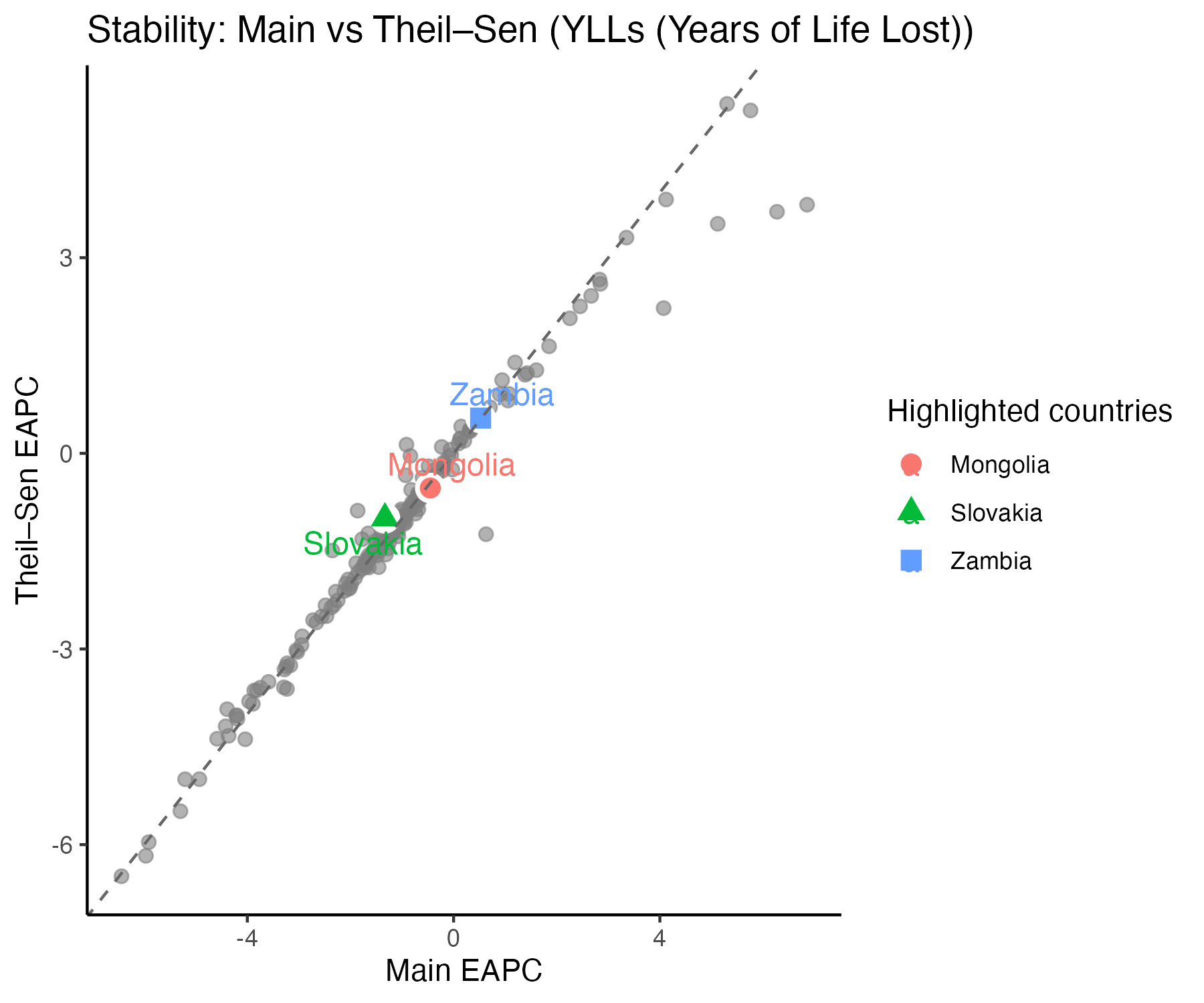

Supplement: Supplementary file 1 [file Data_Sheet_1.zip › stability_YLLs (Years of Life Lost).png]

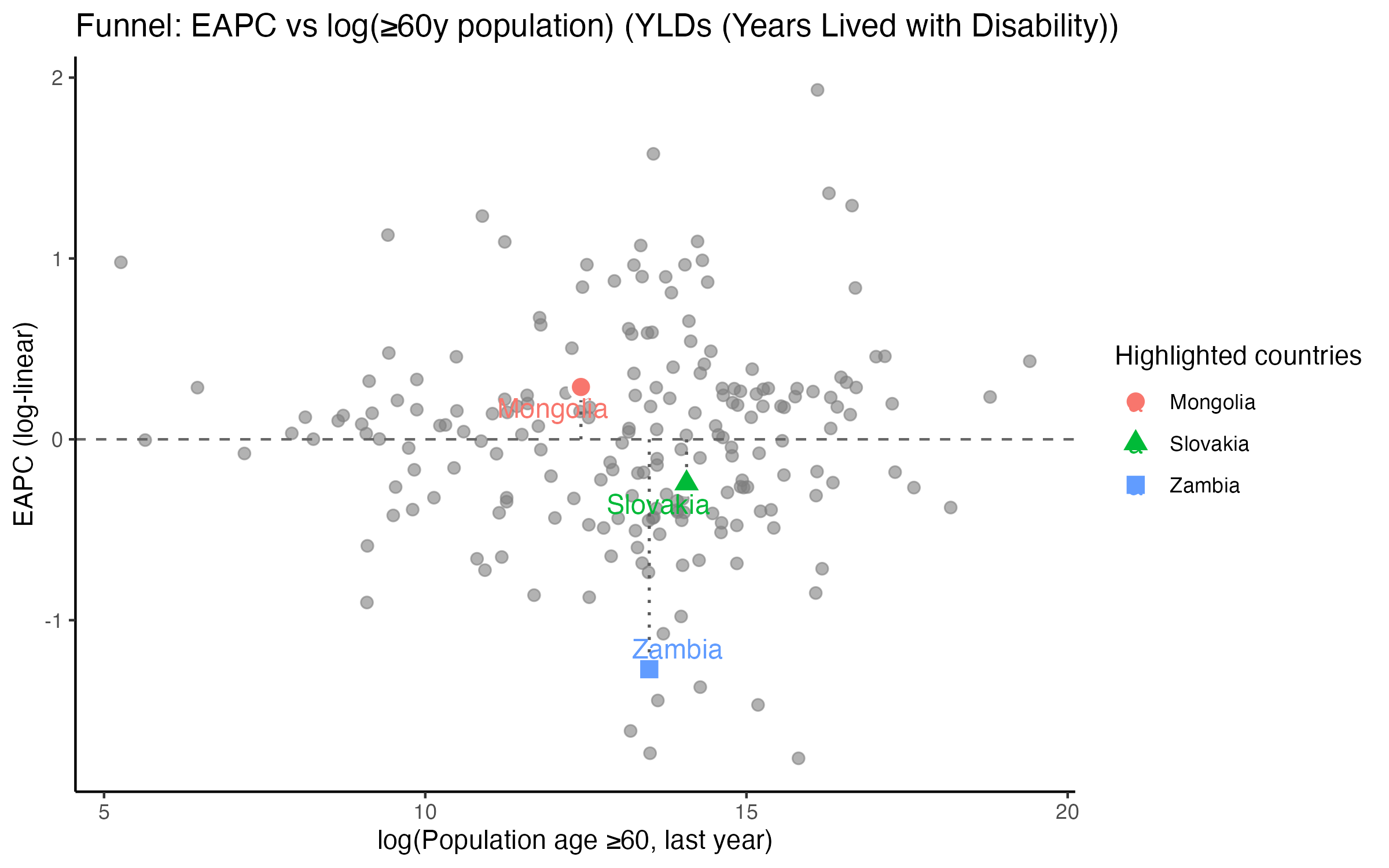

Supplement: Supplementary file 1 [file Data_Sheet_1.zip › funnel_YLDs (Years Lived with Disability).png]

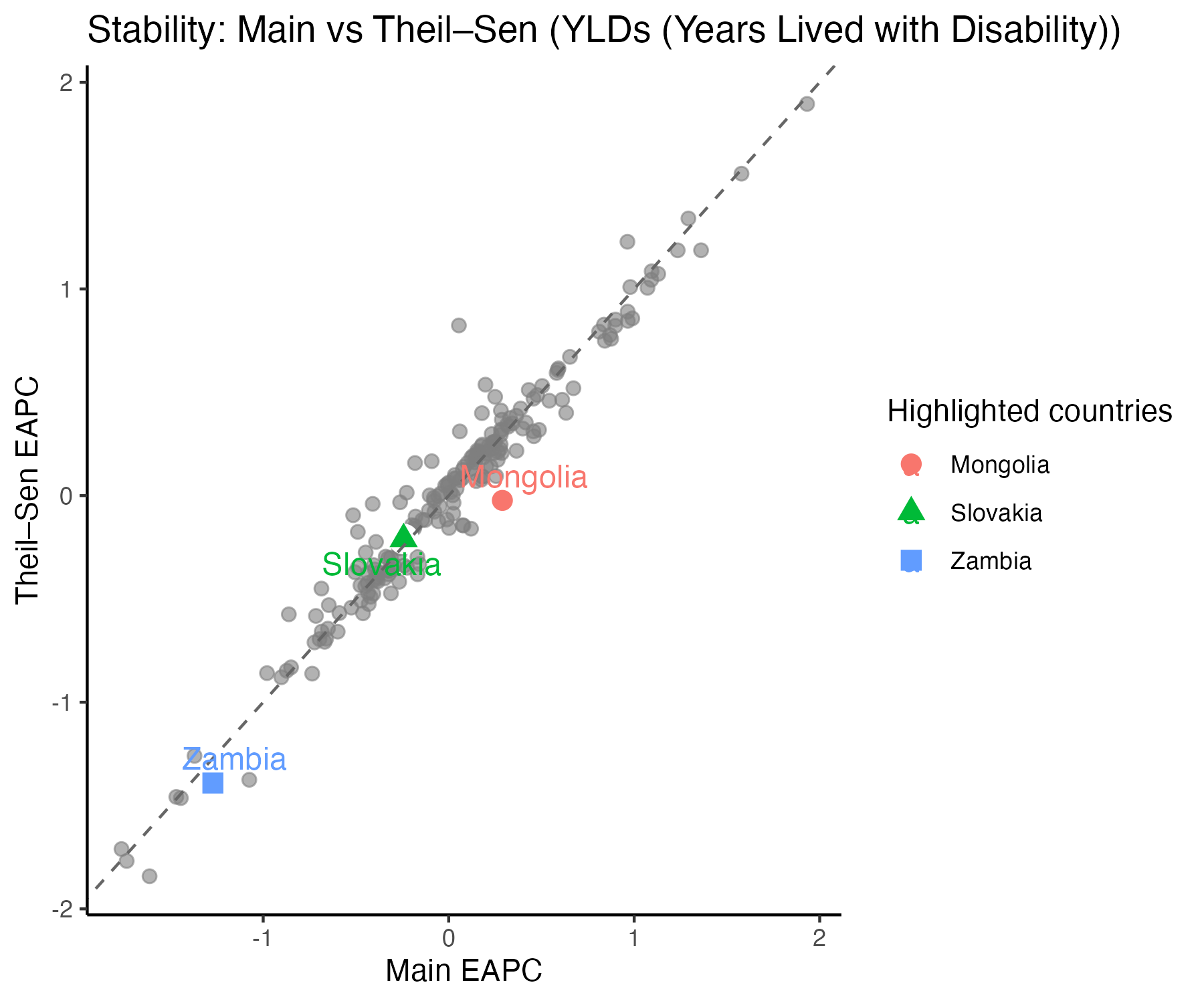

Supplement: Supplementary file 1 [file Data_Sheet_1.zip › stability_YLDs (Years Lived with Disability).png]

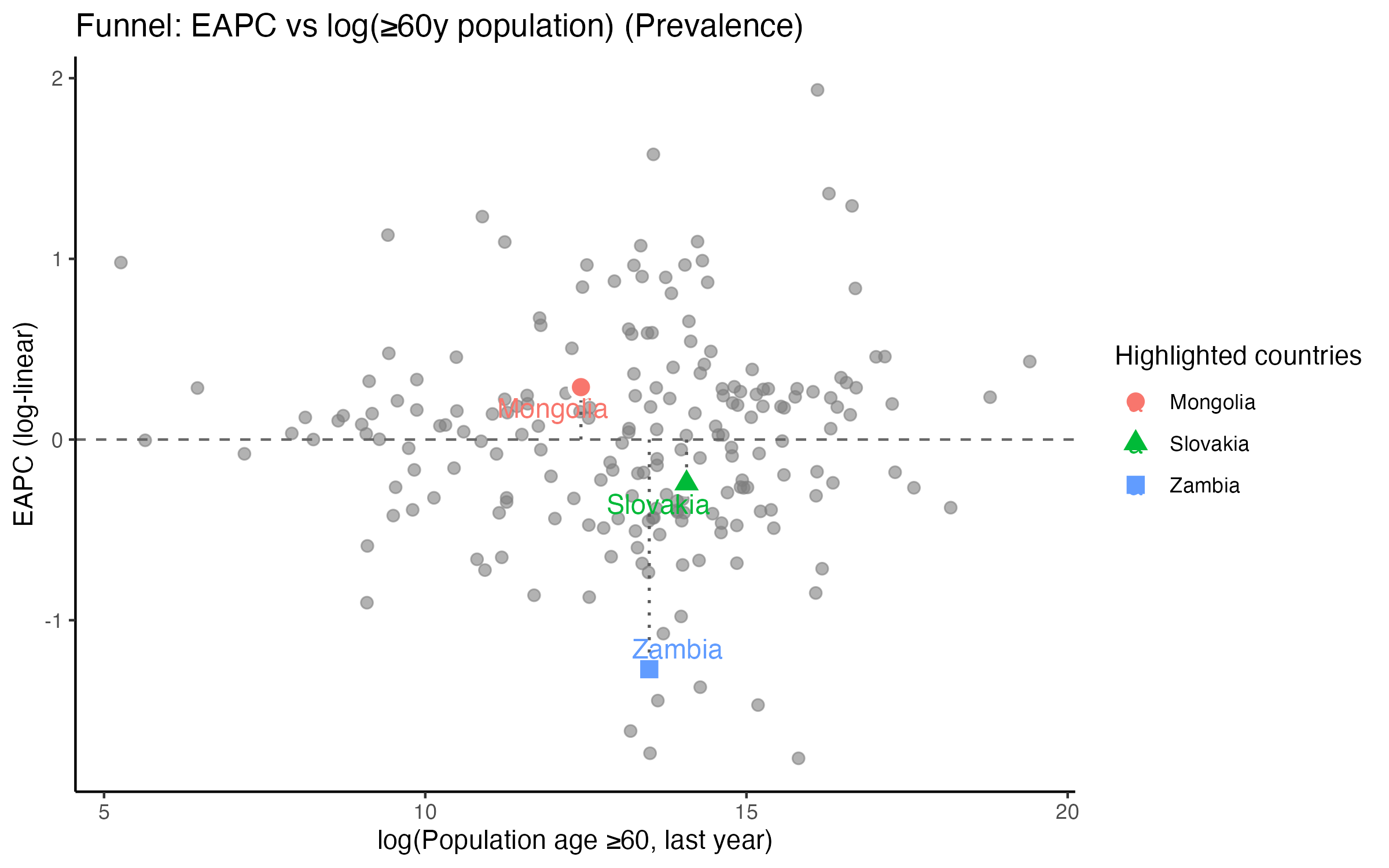

Supplement: Supplementary file 1 [file Data_Sheet_1.zip › funnel_Prevalence.png]

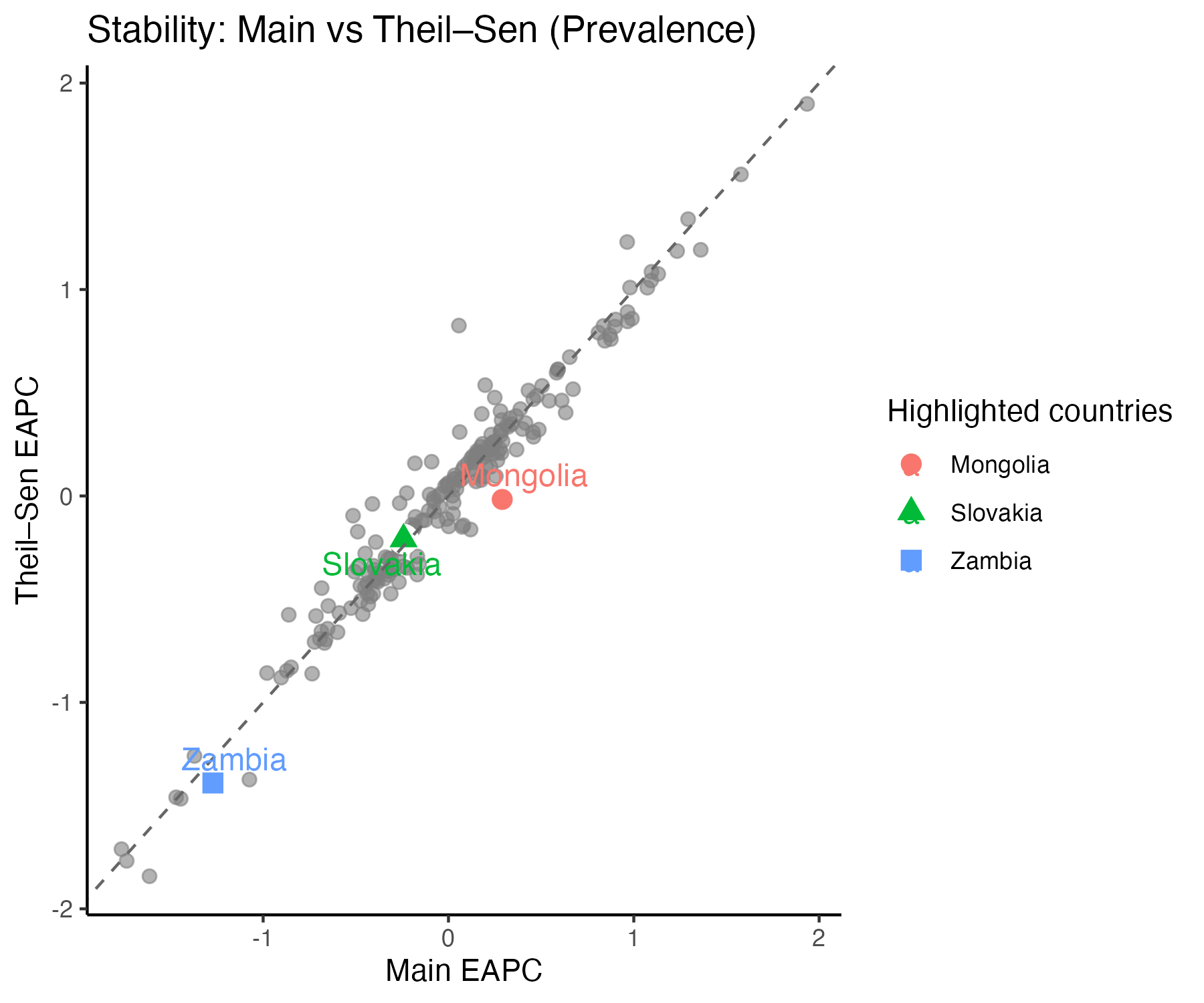

Supplement: Supplementary file 1 [file Data_Sheet_1.zip › stability_Prevalence.png]

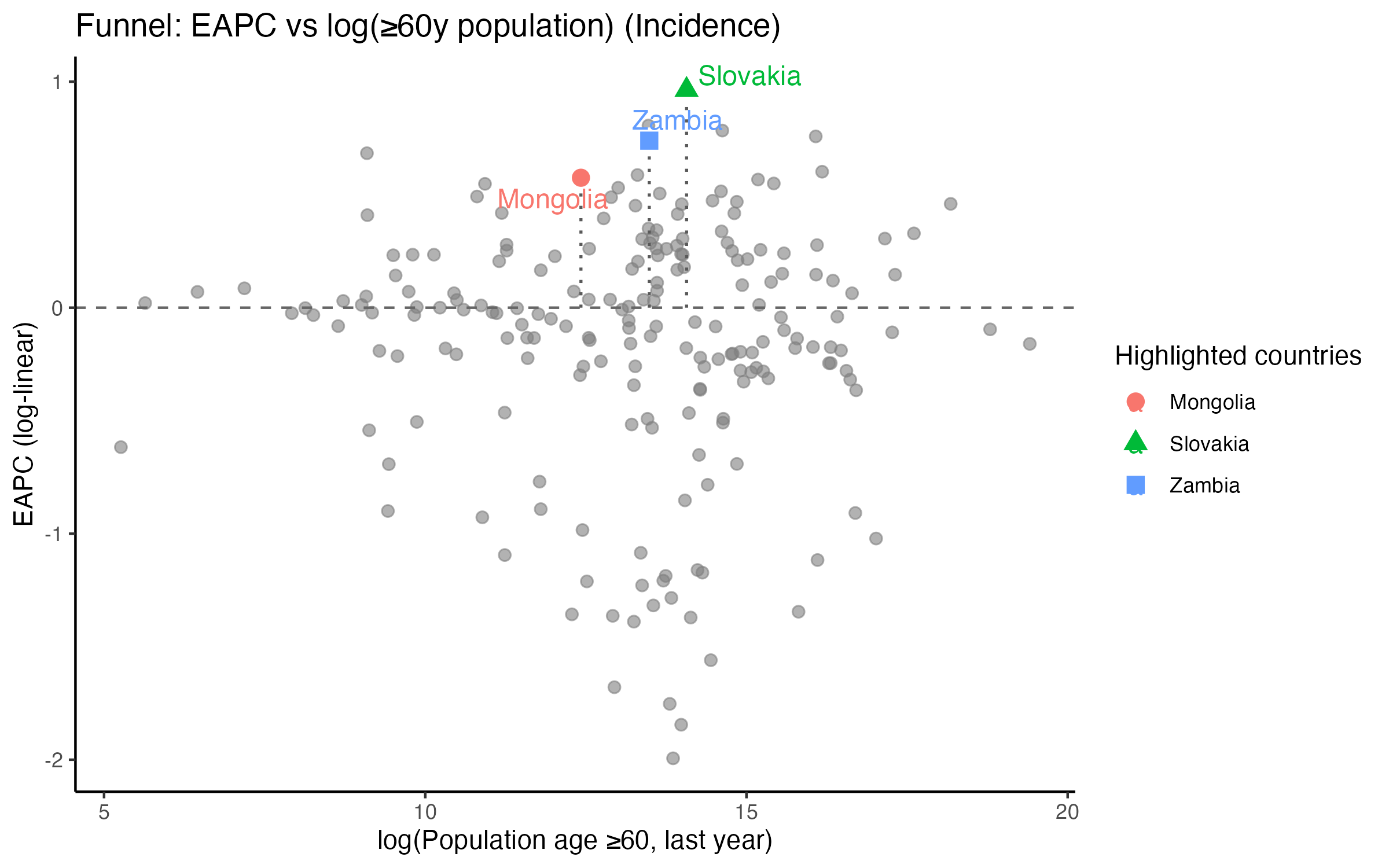

Supplement: Supplementary file 1 [file Data_Sheet_1.zip › funnel_Incidence.png]

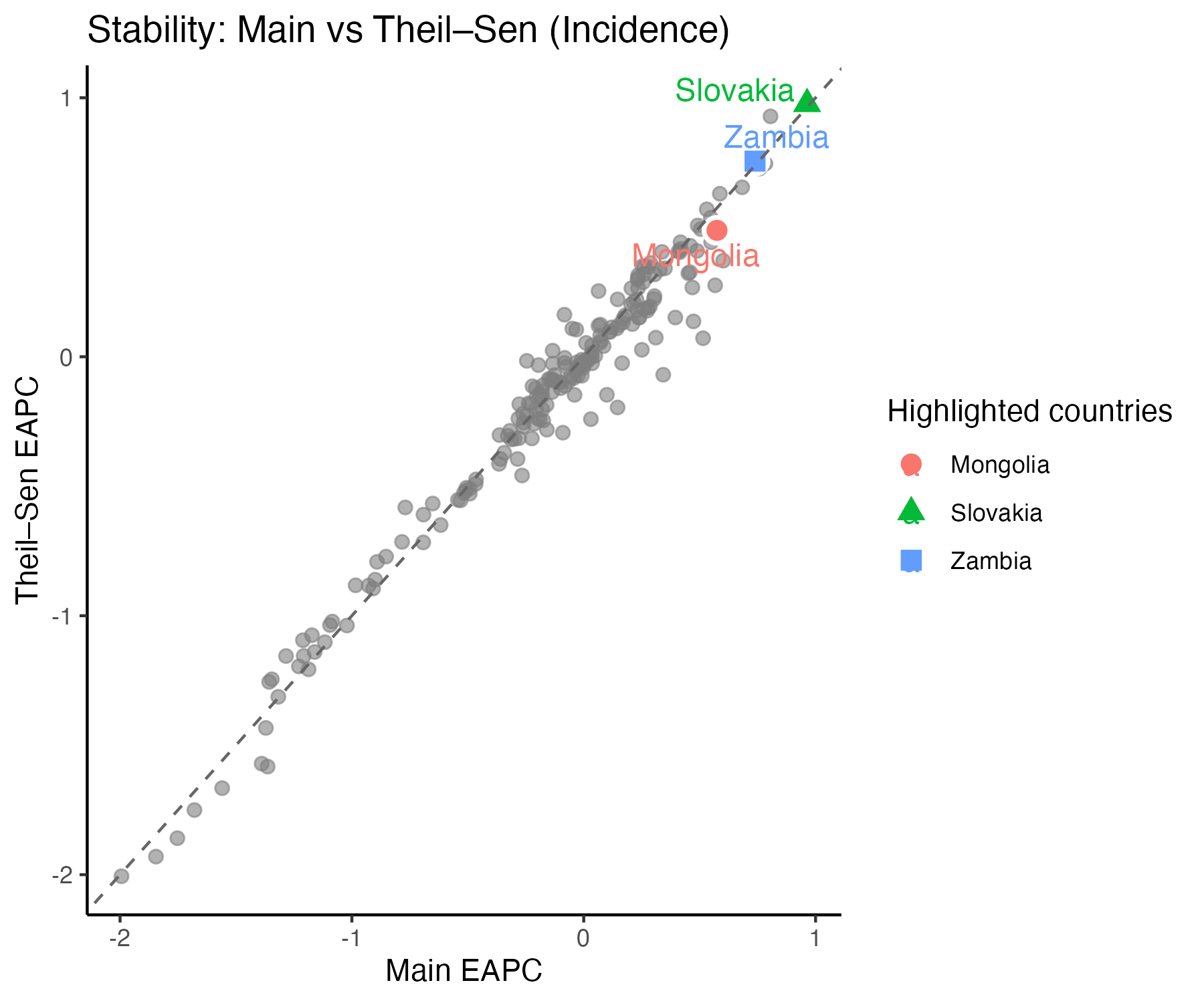

Supplement: Supplementary file 1 [file Data_Sheet_1.zip › stability_Incidence.png]

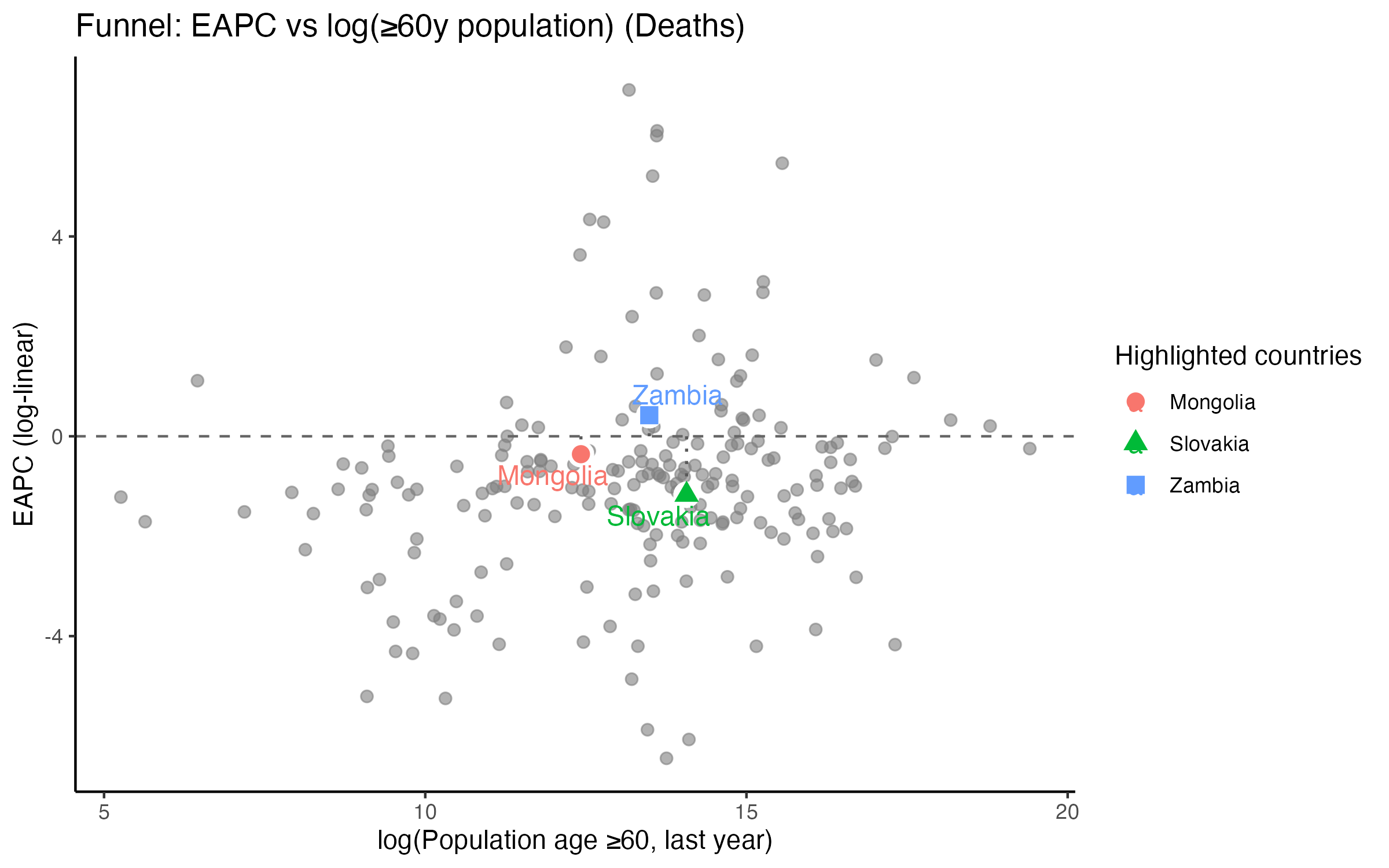

Supplement: Supplementary file 1 [file Data_Sheet_1.zip › funnel_Deaths.png]

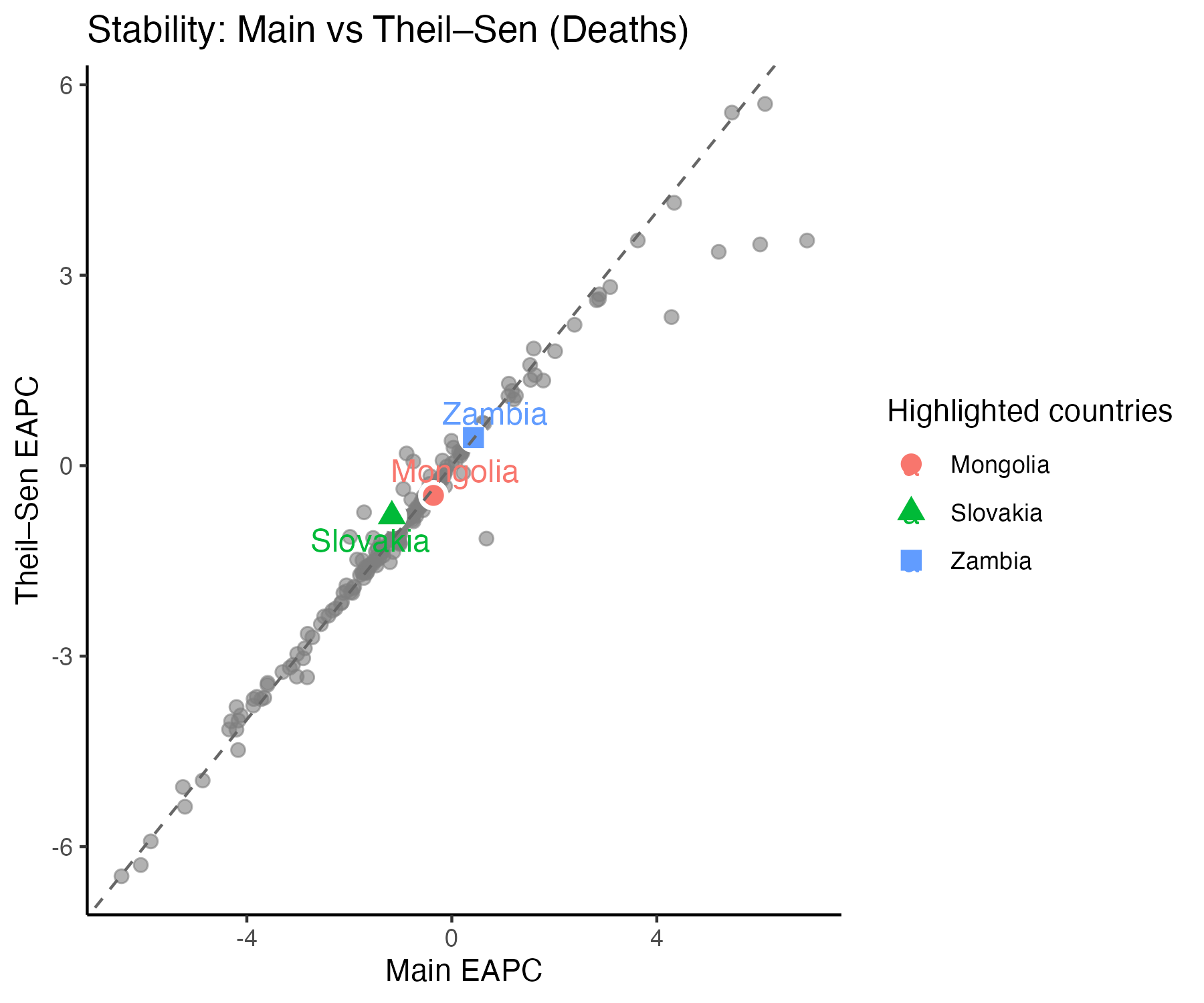

Supplement: Supplementary file 1 [file Data_Sheet_1.zip › stability_Deaths.png]

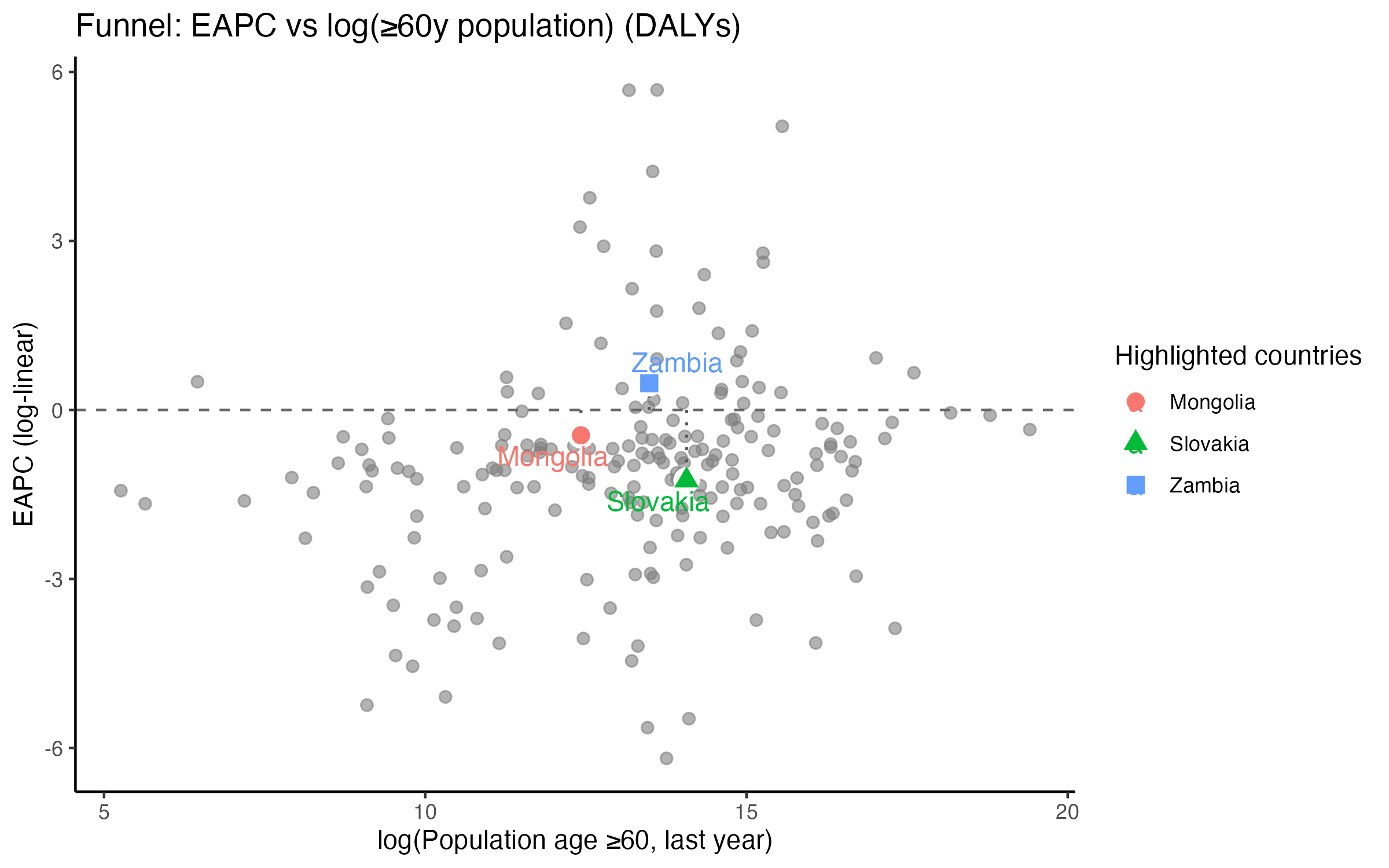

Supplement: Supplementary file 1 [file Data_Sheet_1.zip › funnel_DALYs.png]

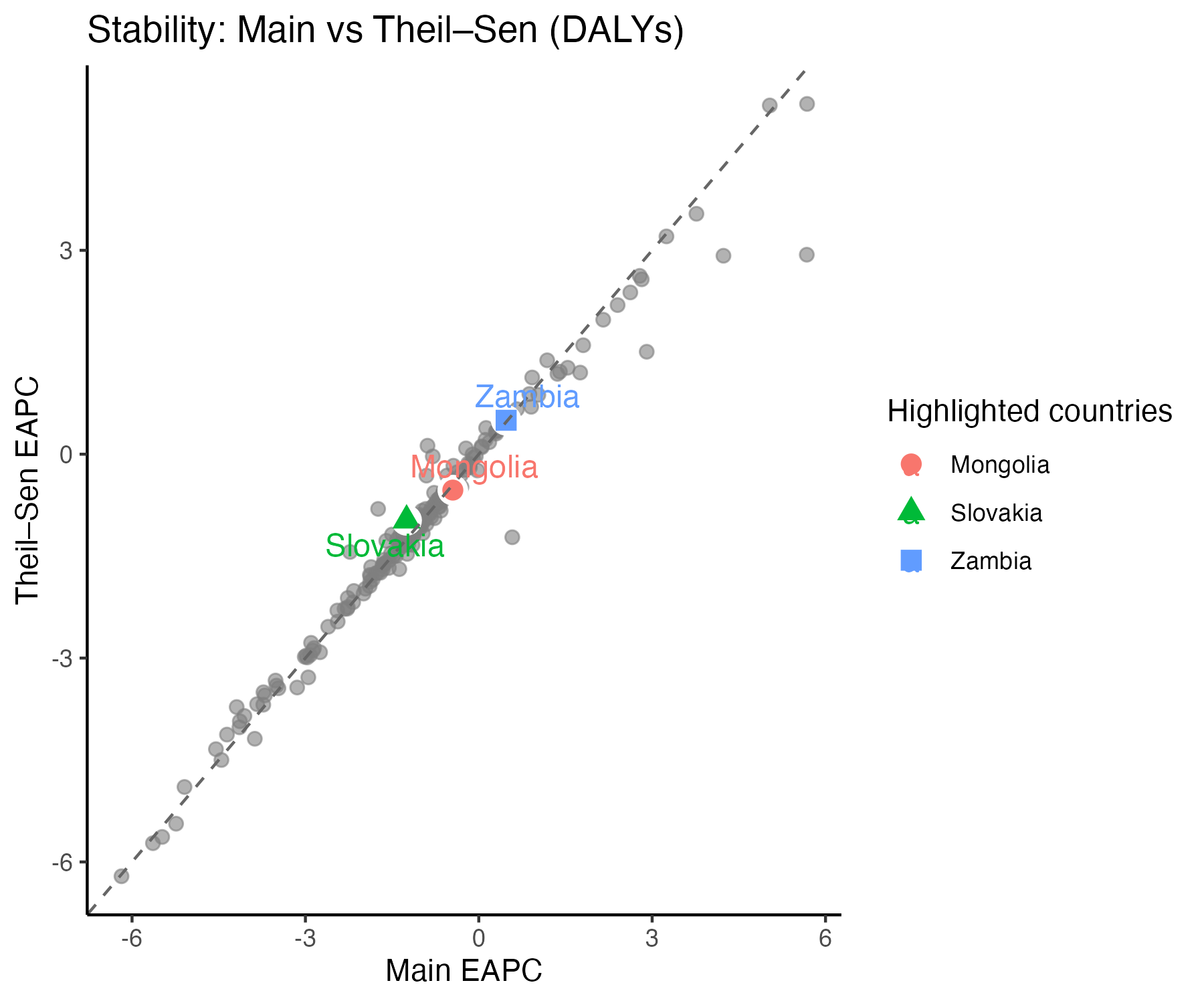

Supplement: Supplementary file 1 [file Data_Sheet_1.zip › stability_DALYs.png]
